# Claudin-2 promotes colorectal cancer liver metastasis and is a biomarker of the replacement type growth pattern

**DOI:** 10.1038/s42003-021-02189-9

**Published:** 2021-06-02

**Authors:** Sébastien Tabariès, Matthew G. Annis, Anthoula Lazaris, Stephanie K. Petrillo, Jennifer Huxham, Amri Abdellatif, Vincent Palmieri, Jaclyn Chabot, Radia M. Johnson, Steven Van Laere, Cornelis Verhoef, Yasmina Hachem, Sara Yumeen, Nicholas Meti, Atilla Omeroglu, Gulbeyaz Altinel, Zu-Hua Gao, Alan S. L. Yu, Dirk J. Grünhagen, Peter Vermeulen, Peter Metrakos, Peter M. Siegel

**Affiliations:** 1 Goodman Cancer Research Centre, McGill University, Montréal, QC Canada; 2 Departments of Medicine, McGill University, Montréal, QC Canada; 3 Department of Surgery, McGill University Health Center, Montréal, QC Canada; 4 Department of Bioinformatics & Computational Biology, Genentech Inc., South San Francisco, CA USA; 5 University of Antwerp, Molecular Imaging, Pathology, Radiotherapy & Oncology (MIPRO), Edegem, Antwerp Belgium; 6 Translational Cancer Research Unit, Oncologisch Centrum GZA, Wilrijk, Antwerp Belgium; 7 Department of Surgical Oncology, Erasmus MC Cancer Institute, Rotterdam, The Netherlands; 8 Department of Pathology, McGill University Health Center, Montréal, QC Canada; 9 Jared Grantham Kidney Institute, University of Kansas Medical Center, Kansas City, KS USA

**Keywords:** Colon cancer, Metastasis

## Abstract

Claudin-2 promotes breast cancer liver metastasis by enabling seeding and early cancer cell survival. We now demonstrate that Claudin-2 is functionally required for colorectal cancer liver metastasis and that Claudin-2 expression in primary colorectal cancers is associated with poor overall and liver metastasis-free survival. We have examined the role of Claudin-2, and other claudin family members, as potential prognostic biomarkers of the desmoplastic and replacement histopathological growth pattern associated with colorectal cancer liver metastases. Immunohistochemical analysis revealed higher Claudin-2 levels in replacement type metastases when compared to those with desmoplastic features. In contrast, Claudin-8 was highly expressed in desmoplastic colorectal cancer liver metastases. Similar observations were made following immunohistochemical staining of patient-derived xenografts (PDXs) that we have established, which faithfully retain the histopathology of desmoplastic or replacement type colorectal cancer liver metastases. We provide evidence that Claudin-2 status in patient-derived extracellular vesicles may serve as a relevant prognostic biomarker to predict whether colorectal cancer patients have developed replacement type liver metastases. Such a biomarker will be a valuable tool in designing optimal treatment strategies to better manage patients with colorectal cancer liver metastases.

## Abstract (web summary)

Tabariès et al. describe that claudin 2 is a promoter of colorectal cancer liver metastasis. Furthermore, high Claudin-2 expression is associated with shorter time to liver-specific recurrence and is a biomarker of replacement type CRC liver metastases.

## Introduction

The liver is a frequent metastatic site for numerous solid cancers; including colorectal, pancreatic, breast, lung, liver, renal cell carcinoma, melanoma, and sarcoma^1–3^. Indeed, ~50% of colorectal cancer (CRC) patients develop liver metastases (LM) and 90% will die as a result of metastatic disease progression^4,5^. When feasible, the current therapy for colorectal cancer liver metastases (CRCLMs) is surgical resection, which improves the 5-year survival rate by 50 percent. While in some cases, surgical resection of CRCLMs can result in a cure^6^, the majority of the patients with CRCLMs (80%) are ineligible for liver resection^7^. Thus, current treatment strategies focus on disease control, prolonging survival, and palliation of symptoms^4^.

Three distinct histopathological growth patterns (HGPs) in CRCLM have been described, which are termed desmoplastic (DHGP)-, pushing (PHGP)-, or replacement (RHGP)-type HGPs^3,8,9^. While the underlying cellular and molecular mechanisms that give rise to these different HGPs remain unknown, each HGP possesses unique histopathological characteristics. In PHGP lesions, which are rare, liver cell plates are pushed aside by growing metastatic cancer cells, becoming compressed and aligned in parallel with the circumference of the metastases at the tumor and liver parenchyma interface. Vascularization occurs by angiogenesis in this growth pattern. There is no evidence of a fibrous rim that surrounds pushing-type metastases and ingrowth of cancer cells into the liver parenchyma is not observed^2^. DHGP metastases are characterized by a desmoplastic rim that separates the CRCLMs from the liver parenchyma, preventing direct contact between the tumor cells and the hepatocytes. DHGP liver metastases engage the uPA–uPAR–PAI-1 proteolytic system, display increased type I and type IV collagen content^2,10–12^ and exhibit extensive stromal remodeling and angiogenesis^3,8,9^. In contrast, RHGP liver metastases arise through the replacement of hepatocytes by cancer cells as the lesion grows. Cancer cells grow around and co-opt existing sinusoidal vasculature of the liver; thus, RHGPs do not rely on angiogenesis to establish a vascular supply^8,9,13^. In up to 90% of the cases, the same histological growth pattern was found in all metastases arising in the same patient^14,15^.

Importantly, these distinct HGPs are associated with different patient prognoses, with a better overall survival observed in patients that develop CRCLMs with desmoplastic features^8,16–18^. It has recently been suggested that angiogenic DHGPs respond best to treatment regimens that incorporate chemotherapy in combination with antiangiogenic agents (bevacizumab)^8^. In contrast, patients with RHGP metastases that rely on vessel co-option respond poorly to chemotherapy plus antiangiogenic therapy^8^. It has recently been demonstrated that 19% of chemo-naïve patients with CRC liver metastases present with desmoplastic lesions. The proportion of DHGP liver metastases increases to 30% in patients treated with neoadjuvant chemotherapy^16^.

Claudins are tight junctions constituents that play complex roles during the metastatic process^19–23^. Claudin-2 is expressed in breast cancer liver metastases^24,25^ and high Claudin-2 levels in primary breast tumors are specifically associated with the formation of liver metastases^26^. Claudin-2 facilitates breast cancer cell interactions with the extracellular matrix and hepatocytes, which increases liver metastasis^24,27^. More recently, the PDZ-binding motif located at the C-terminus of Claudin-2 was shown to be important for its pro-metastatic functions by facilitating interactions with Afadin^25^. Claudin-2 levels are also increased in CRC and gastric cancers, two solid cancers that metastasize to the liver^28–33^. Claudin-2 promotes protumorigenic phenotypes, including increasing anchorage-independent growth of colorectal cancer cells^30,34–36^. Furthermore, Claudin-2 levels in CRC cells confers chemo-resistance in vitro and elevated Claudin-2 expression in CRC-associated fibroblasts is correlated with shorter survival in treated metastatic CRC patients^34,37^.

In this study, we explore the functional role of Claudin-2 in promoting CRC liver metastasis and evaluate the role of claudin family members as pathological and prognostic markers of DHGP or RHGP CRCLM.

## Results

### Claudin-2 promotes the efficient formation of CRC liver metastases

**Fig. 1:**
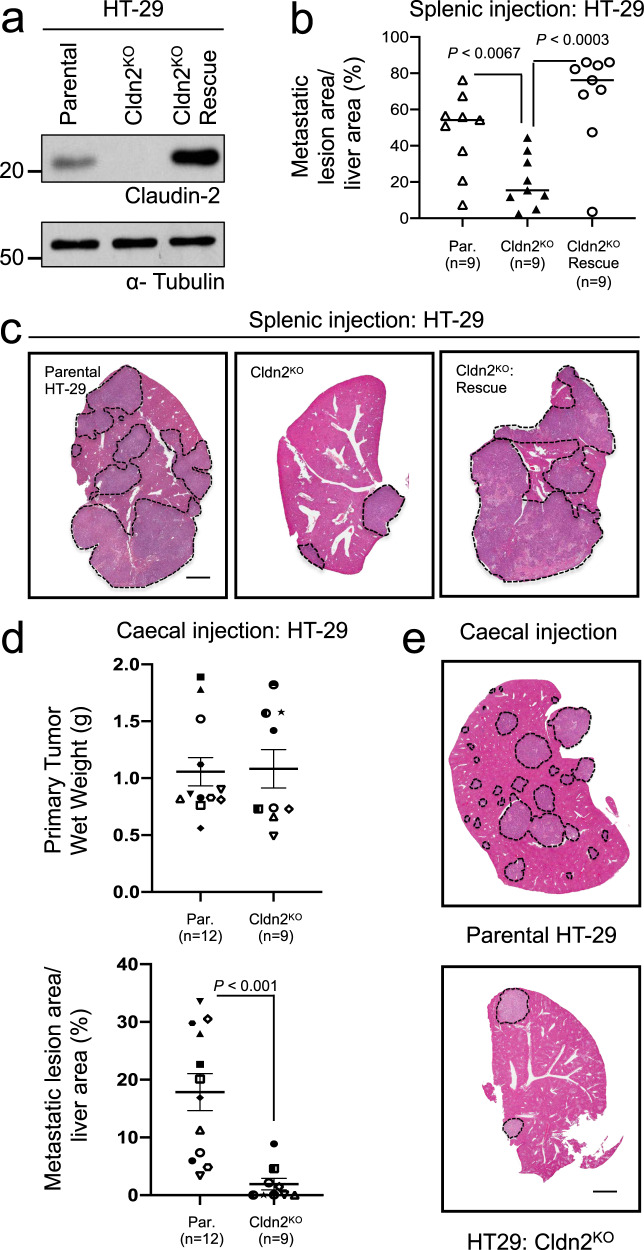
Claudin-2 promotes efficient colorectal cancer liver metastasis. **a** Immunoblot analysis of Claudin-2 expression in parental HT-29 cells, HT-29 cells with Claudin-2 knockout (Cldn2^KO^) and HT-29 cells lacking endogenous Claudin-2 that were engineered to re-express Claudin-2 (Cldn2^KO^ rescue). Total cell lysates were blotted for α-Tubulin as a loading control. **b** Quantification of the metastatic burden (lesion area/tissue area) within the cardiac liver lobe following splenic injection. **c** Representative hematoxylin & eosin (H&E) images of the cardiac liver lobe are shown for mice injected (splenic) with the indicated cell populations. **d** Quantification of the primary tumor burden (wet weight) and metastatic burden (lesion area/tissue area) within the cardiac liver lobe following caecal injection. **e** Representative H&E images of the cardiac liver lobe are shown for mice injected (caecal) with the indicated cell populations. **c**, **e** Dotted lines circumscribe colorectal cancer metastatic lesions within the liver. Scale bar, 2 mm and applies to all panels. Data are presented as the mean ± standard error (SE).

Claudin-2 enhances breast cancer metastasis to the liver^24,25,27,38^ and increases the tumorigenicity of colorectal cancer cells^30,34–36^. To extend these observations, we assessed the ability of Claudin-2 to promote colorectal cancer liver metastases. To generate a Claudin-2 knockout in human HT-29 CRC, independent clones were first screened by immunoblot analysis to identify those with Claudin-2 loss and a pool of three Claudin-2 knockout clones was generated (Cldn2^KO^; Fig. 1a). To confirm that any observed phenotypes in Cldn2^KO^ CRC cells were not the result of off-target effects, we performed a rescue of Claudin-2 expression (Cldn2^KO^ Rescue; Fig. 1a). HT-29 cells lacking Claudin-2 failed to efficiently form liver metastases, exhibiting a 2.37-fold reduction in liver-metastatic burden when compared to the parental controls (Fig. 1b, c). Expression of Claudin-2 resulted in a 3.36-fold increase in metastatic burden when compared to Cldn2^KO^ cells (Fig. 1b, c). We next determined whether Claudin-2 also promotes spontaneous CRC liver metastasis. While no significant change in primary tumor growth following caecal injection was observed between parental and Cldn2^KO^ HT-29 cells (Fig. 1d), CRC cells lacking Claudin-2 demonstrated an average 9.3-fold decrease in their spontaneous liver-metastatic potential (Fig. 1d, e). To assess potential correlations between Claudin-2 expression levels in primary CRC tumors (Fig. 1d) and the extent of liver metastasis, we selected two primary tumors from mice that developed high, intermediate and low liver-metastatic burden following intracaecal injection of HT-29 cells. We observed similar levels of Claudin-2 immunohistochemical staining across all three groups, with highly variable staining within each group. However, no correlation was evident between Claudin-2 levels in the HT-29 CRC tumors and the degree of spontaneous liver metastasis (Supplementary Fig. 1).

To examine whether Claudin-2 can enhance the ability of CRC cells to form liver metastases, we stably overexpressed Claudin-2 in SW403 cells that lack endogenous Claudin-2 (Supplementary Fig. 2a). Following splenic injection, Claudin-2 overexpressing cells (Cldn2^OE^) showed a higher tumor burden in the liver when compared with their respective controls (Supplementary Fig. 2b, c). Thus, lesions formed by Claudin-2 overexpressing cells occupied 2.38 times more liver area when compared with control cells (Supplementary Fig. 2b, c). Together, these data suggest that Claudin-2 is a critical mediator promoting colorectal cancer liver metastasis.

### The Claudin-2 PDZ-binding motif contributes to the formation of CRC liver metastases

To assess the importance of the PDZ-binding motif for Claudin-2-mediated CRC liver metastasis, we engineered HA-tagged versions of both wild-type Claudin-2 and a mutant lacking the C-terminal PDZ-binding motif in HT-29 CRC cells (Supplementary Fig. 3a)^25^. Immunoblot analysis with an anti-Claudin-2 antibody demonstrated that endogenous Claudin-2 was indeed lost and confirmed that untagged wild-type Claudin-2 was expressed (Supplementary Fig. 3b). As previously reported^23^, removal of the PDZ-binding domain abrogates detection with the anti-Claudin-2 antibody used in these experiments (Supplementary Fig. 3b). Immunoblotting with an anti-HA-tag antibody confirmed expression of the HA-tagged constructs (wild-type Claudin-2 and the the ΔPDZ BD mutant) (Supplementary Fig. 3b). Following splenic injection, mice receiving pooled populations expressing wild-type Claudin-2 (Cldn2^KO^: WT, Cldn2^KO^: HA-WT) developed significant and comparable metastatic burden (Fig. 1b; Supplementary Fig. 3c, d), whereas the cohort injected with Cldn2^KO^: ΔPDZ BD expressing CRC cells exhibited reduced liver-metastatic burden (Supplementary Fig. 3c, d). The ability of wild-type Claudin-2 expressing CRC cells to form liver metastases was increased 2-fold compared to the Claudin-2 ΔPDZ BD expressing population (Supplementary Fig. 3c, d). We next determined whether the PDZ-binding motif of Claudin-2 was required for the formation of spontaneous CRC liver metastases. While no significant change in primary tumor growth following caecal injection was observed in mice injected with Cldn2^KO^: WT, Cldn2^KO^: HA-WT, and Cldn2^KO^: HA-ΔPDZ HT-29 cells (Supplementary Fig. 3e, f), animals injected with CRC cells expressing Claudin-2 lacking the PDZ-binding domain exhibited an average 9.4-fold decrease in spontaneous liver-metastatic burden relative to mice injected with CRC cells expressing wild-type Claudin-2 (Supplementary Fig. 3e, f). These data demonstrate that the PDZ-binding motif within Claudin-2 is required for efficient colorectal cancer liver metastasis.

### Loss of Claudin-2 in human CRC cancer cells is associated with reduced formation of lung metastases

To determine whether the metastasis-promoting effects of Claudin-2 in HT-29 were restricted to the liver, we assessed the impact of Claudin-2 loss on CRC lung metastasis. It should be noted that HT-29 cells give rise to a very low metastatic burden in the lungs following tail vein injection. Despite this caveat, tail vein injections revealed that Claudin-2-deficient cells (Cldn2^KO^) exhibited a 10.1-fold reduction in lung-metastatic burden when compared with parental controls expressing endogenous Claudin-2 (Supplementary Fig. 4a, b). Furthermore, animals injected with Cldn2^KO^: HA-ΔPDZ HT-29 cells exhibited an average 9.9-fold decrease in lung-metastatic burden when compared to mice injected with Cldn2^KO^: HA-WT cells (Supplementary Fig. 4a, b). Thus, Claudin-2 promotes the formation of CRC metastases in both the liver and lung, which requires the Claudin-2 PDZ-binding motif.

### Claudin-2 promotes colorectal cancer cell adhesion to hepatocytes

Claudin-2 promotes breast cancer metastasis to the liver, in part, by facilitating cancer cell interaction with hepatocytes^27^. Thus, we assessed whether Claudin-2 could also facilitate the ability of colorectal cancer cells to adhere to primary hepatocytes. Parental HT-29 cells display a 2.7-fold higher ability to adhere to primary hepatocyte monolayers when compared to Claudin-2 deficient cancer cells (Supplementary Fig. 5a, b). Expression of either Cldn2^KO^: HA-WT or Cldn2^KO^: HA-ΔPDZ completely rescued the ability of HT-29 cells to interact with hepatocytes relative to populations lacking Claudin-2. Indeed, Cldn2^KO^: HA-WT or Cldn2^KO^: HA-ΔPDZ expressing HT-29 cells exhibited a 2.6–2.7-fold increase in hepatocyte adhesion when compared to Cldn2^KO^: VC cells (Supplementary Fig. 5a, b), demonstrating that the PDZ-binding domain is dispensable for this phenotype.

We next assessed if the ability of colorectal cancer cells to adhere to hepatocytes was mediated through a *trans*-homotypic interaction between Claudin-2 molecules expressed in both cell types, as previously demonstrated for breast cancer cells^27^. Adhesion of HT-29 colorectal cancer cells display a 2.2-fold higher ability to adhere to primary Claudin-2-expressing hepatocytes when compared to Claudin-2-deficient hepatocytes (Supplementary Fig. 5c, d). Taken together, our results demonstrate that colorectal cancer cells expressing Claudin-2 interact with resident hepatocytes through the formation of *trans*-homotypic Claudin-2/Claudin-2 interactions.

### Claudin-2 expression in human primary colorectal cancers predicts liver metastasis

Claudin-2 expression in primary breast tumors is prognostic for the development of liver metastases^25,26^. To extend this observation to CRC, we performed immunohistochemical staining for Claudin-2 on 87 CRC patient primary tumors. We observed that Claudin-2 levels were significantly elevated in primary tumors from CRC patients that relapsed to the liver within 5 years compared to those patients that did not develop liver metastases within 5 years of diagnosis (Supplementary Fig. 6a, b). To extend these analyses, we also assessed Claudin-2 expression in 22 primary colorectal cancer primary tumors and their matched liver metastases. In the majority of cases, Claudin-2 expression levels were already high in the primary tumors and no further enrichment in liver metastases was observed (Supplementary Fig. 6c). Patients with high Claudin-2 expression experienced poor overall and relapse-free survival compared to patients with low Claudin-2 expression, with the former reaching statistical significance (Supplementary Fig. 6d, e). Together, this indicates that high Claudin-2 expression in CRC primary tumors is prognostic for the development of liver metastases in CRC patients.

### Claudin-2 expression in human colorectal cancer is associated with replacement-type liver metastasis

**Fig. 2:**
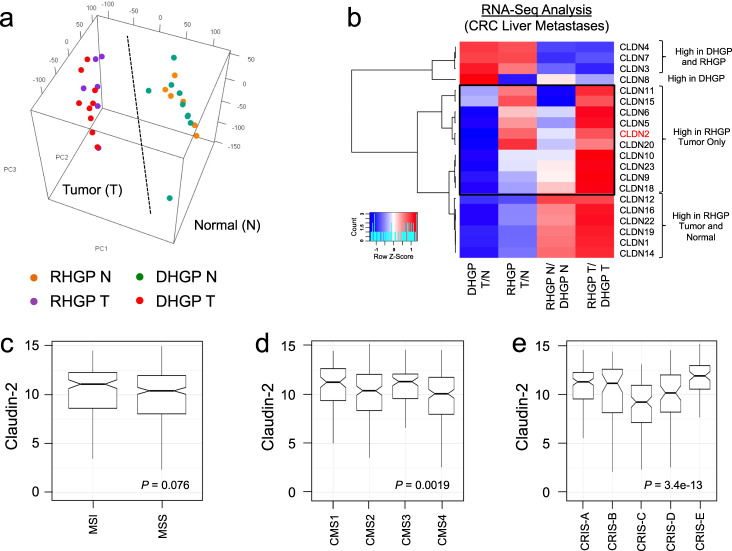
Distinct *Cldn* mRNA expression profiles are associated with desmoplastic and replacement-type CRCLMs. **a** PCA analysis revealed that all tumor or normal samples clustered together. **b** Gene expression heatmaps of *claudin* family members in the dissected samples. A blue to red gradient represents low to high gene expression levels. RHGP Replacement Histopathological Growth Pattern, DHGP Desmoplastic Histopathological Growth Pattern. **c**–**e** Differences in CLDN2 expression (*Y*-axis) between 377 colon adenocarcinoma tissue samples, from the PCAWG dataset, classified according to the MSI (**c**), CMS (**d**), and CRIS (**e**) subtypes are shown in notched boxplot format. Boxes represent the 25th (lower border), median, and 75th (higher border) percentile of the CLDN2 expression distribution in each subgroup, and whiskers extend to the 5th (bottom) and 95th (top) percentile. The notches represent the 95% confidence interval around the median. Outliers are shown as dots. *P*-value resulting from comparing the distributions with a Wilcoxon signed rank test are shown.

We next examined the relationship between Claudin expression and the CRCLM HGPs^9^. Breast cancer liver metastases, which rely on Claudin-2 for their efficient formation, are characterized predominately as the RHGP type^8,9^. RNA-Seq analyses were performed on CRCLMs from patients treated at the McGill University Health Centre (MUHC) to assess the expression levels of Claudin family members (MUHC Cohort)^39^. RNA was isolated from macro-dissected material containing the liver-metastatic lesion (T) or normal adjacent liver (N) from 9 DHGP or 6 RHGP chemo-naïve liver samples. PCA analysis revealed that all tumor or normal samples clustered together (Fig. 2a). For a given HGP, tumor samples were compared to their corresponding normal adjacent counterpart (DHGP T/N or RHGP T/N). In parallel, for a given sample type, the RHGP samples were compared to the DHGP samples (RHGP N/DHGP N or RHGP T/DHGP T). From these analyses, we observed that claudin family members could be clustered into four categories: (1) Claudins with high expression levels in both HGPs (*Cldn-4, -7 and -3*); (2) Claudins with higher expression levels in both the tumor or normal adjacent RHGP samples compared to DHGP samples (*Cldn-12, -16, -22, -19, -1,* and *-14*); (3) Claudins with higher expression only in DHGP tumor samples compared to RHGP tumor samples (*Cldn-8*); and (4) Claudins with higher expression only in RHGP tumor samples compared to DHGP tumor samples (*Cldn-11, -15, -6, -5, -2, -20, -10, -23, -9,* and *-18*) (Fig. 2b). These data demonstrate that claudin expression level varies according to the CRCLM HGP, with Claudin-8 being specifically expressed at higher levels in DHGP and Claudin-2 as one of the claudins specifically expressed at higher levels in RHGP.

Interrogating the Pan Cancer Analysis of Whole Genomes (PCAWG) dataset^40^, we observed, among 377 colon adenocarcinoma tissue samples, a trend for the enrichment of Claudin-2 expression in samples associated with a microsatellite instablility (MSI) phenotype relative to the microsatellite stable subtype (MSS) (Fig. 2c)^41^. With respect to the consensus molecular subtypes (CMS) in CRC^42^, elevated Claudin-2 expression is associated with the CMS1 (hypermutated, microsatellite unstable and strong immune activation) and CMS3 (epithelial and evident metabolic dysregulation) subtypes compared to CMS2 (marked WNT and MYC signaling activation) and CMS4 (prominent transforming growth factor–β activation, stromal invasion, and angiogenesis) (Fig. 2d)^42^. We also examined Claudin-2 expression in the CRC intrinsic subtype (CRIS) stratification system^43^. Higher Claudin-2 expression was observed in the CRIS-A (mucinous, glycolytic, MSI, KRAS mutation), CRIS-B (TGF-β pathway activity, epithelial–mesenchymal transition, poor prognosis), and CRIS-E (Paneth cell-like phenotype, TP53 mutations) subtypes (Fig. 2e). The lowest Claudin-2 levels were associated with the CRIS-C (elevated EGFR signaling, sensitivity to EGFR inhibitors) subtype (Fig. 2e) and intermediate Claudin-2 levels were observed in the CRIS-D (WNT activation, IGF2 expression/amplification) subtypes^43^. Finally, we examined correlations between Claudin-2 expression and the mutational status of oncogenes/tumor suppressors associated with CRC. A correlation between elevated Claudin-2 expression and KRAS or PIK3CA mutations was observed in primary CRC specimens (Supplementary Table 1).

### Claudin-8 is enriched in DHGP lesions while Claudin-2 is enriched in RHGP liver metastases from colorectal cancer patients

**Fig. 3:**
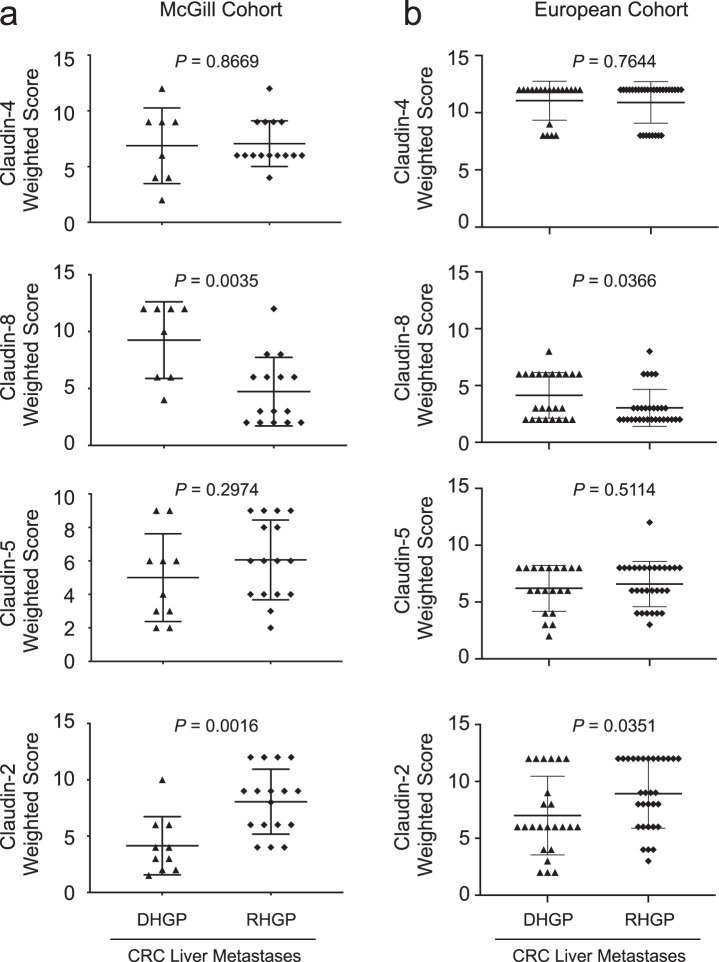
Claudin-2 protein is enriched in the replacement-type lesions while higher Claudin-8 expression is associated with desmoplastic-type liver metastases. **a, b** Paraffin embedded sections from colorectal liver specimens were subjected to immunohistochemical staining with anti-Claudin-4, anti-Claudin-8, anti-Claudin-5, or anti-Claudin-2 antibodies in both the McGill (**a**) or European (**b**) cohorts. Claudin-8 expression is enriched in the desmoplastic-type lesions (DHGP). In contrast, Claudin-2 expression is increased in the replacement-type liver metastases (RHGP). Scoring of IHC staining was performed by two independent pathologists (AO, GA). Data are presented as the mean ± SE.

We next examined the potential relevance of Claudin protein expression for the pathological assessment of CRCLM HGPs using clinical samples from patients with either DHGP (*n* = 8) or RHGP (*n* = 15) liver metastases. In agreement with the RNA-Seq data, immunohistochemical staining (IHC) for Claudin-4 revealed no differences between the DHGPs and RHGPs (Fig. 3a, Supplementary Fig. 7a); whereas, IHC for Claudin-8 demonstrated an enrichment in DHGP lesions (Fig. 3a, Supplementary Fig. 7a). While only a trend for elevated Claudin-5 expression was observed in RHGP lesions (Fig. 3a; Supplementary Fig. 7a), Claudin-2 expression was noticeably enriched in RHGP compared to DHGP CRCLMs (Fig. 3a, Supplementary Fig. 7a). These observations were further corroborated using a validation cohort of samples composed of 24 DHGP and 31 RHGP samples (European Cohort) (Fig. 3b, Supplementary Fig. 7b). Thus, our results argue that differential claudin expression in CRC liver metastasis may be useful for the pathological assessment of CRC liver metastasis HGPs.

### Patient derived xenografts (PDXs) from CRCLMs recapitulate desmoplastic- and replacement-type growth patterns

**Fig. 4:**
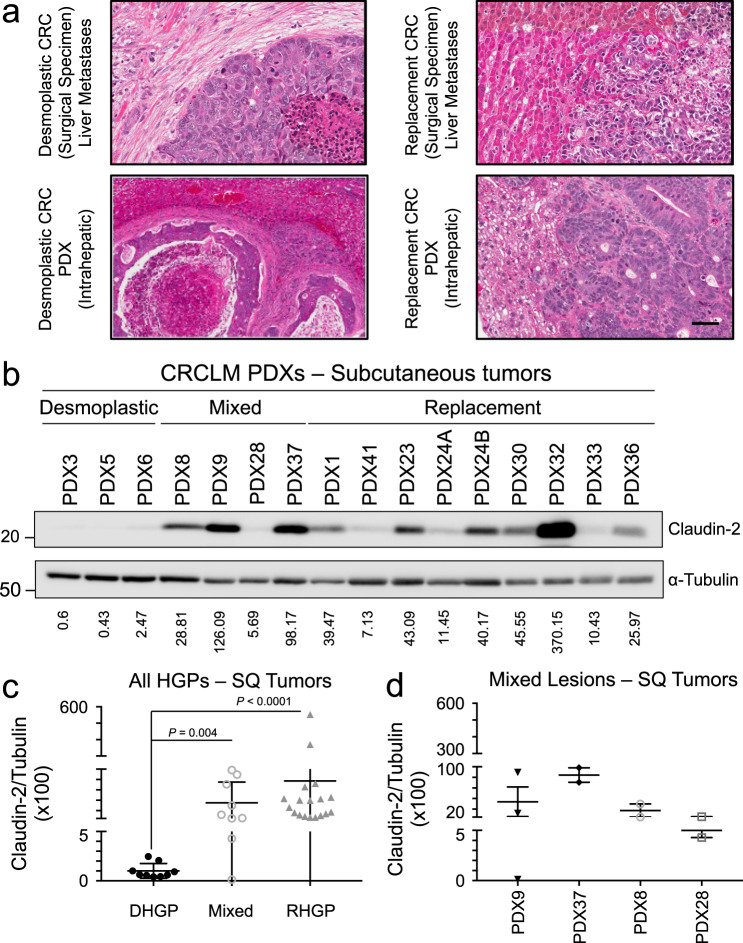
PDX-derived models for both replacement- and desmoplastic-type liver metastases. **a** H&E stains of clinical CRC liver metastases illustrating the desmoplastic and replacement-type histopathologies (top panels) compared to matched PDX samples following intrahepatic transplantation of colorectal cancer patient liver metastasis tissue (bottom panels). The scale bar = 50 μm and applies to all panels. **b** Representative immunoblot analysis of Claudin-2 expression in subcutaneous tumor lysates from PDXs. As a loading control, total cell lysates were blotted for α-Tubulin. **c** Claudin-2 expression is elevated in subcutaneous tumors derived from replacement-type metastases or those arising from mixed lesions containing replacement-type features. **d** Detailed assessment of the Claudin-2/Tubulin ratio in the mixed lesions. The ratio of Claudin-2 to α-Tubulin was measured using an Odyssey infrared imaging system and are indicated in each panel (**b**–**d**). Data are presented as the mean ± SE.

To further characterize the potential role of Claudins in CRCLM HGPs, we successfully established both subcutaneous and intrahepatic patient-derived xenografts (PDXs) using resected material from CRCLM patients diagnosed with either DHGP or RHGP-type liver metastases. Following intrahepatic transplantation of human material in Scid-Beige mice, DHGP donor metastases produced desmoplastic-type lesions while RHGP donor metastases gave rise to replacement-type lesions (Fig. 4a). We have established 12 RHGP (success rate of 86%) and 7 DHGP (success rate of 64%) type lesions as subcutaneous PDXs as well as 8 RHGP (success rate of 73%) and 7 DHGP (success rate of 78%) type CRCLMs as intrahepatic lesions (Supplementary Table 2). Of importance, the observed HGP was conserved following serial passage by intrahepatic transplantation (Supplementary Table 2). The human origin of tumor cells in our PDX-derived intrahepatic lesion was further validated by immunohistochemical staining against cytokeratin 20 (CK20) (Supplementary Fig. 8).

### Claudin-2 is enriched in RHGP PDX models derived from CRC patients with RHGP liver metastases

We next assessed if our previous observations demonstrating an enrichment of Claudin-2 expression in RHGP CRCLMs was maintained in subcutaneous PDX models that retained features of replacement HGP metastases. Indeed, the Claudin-2/Tubulin ratio was higher in RHGP derived PDXs (average of 65.93; ranging from 7.13 to 370.15) compared to DHGP PDXs (average of 1.17; ranging from 0.43 to 2.47) (Fig. 4b, c). In addition, PDXs derived from mixed type CRCLMs (metastases that possess features of more than one HGP) were also established (Supplementary Table 2). The mixed metastases demonstrated an intermediate Claudin-2/Tubulin ratio (Fig. 4c). A detailed assessment of the mixed lesions revealed that the Claudin-2/Tubulin ratio was positively correlated with lesions that have predominantly RHGP features (Fig. 4d; Supplementary Table 2). In contrast, the ratio of Claudin-8 to Tubulin was higher in DHGP derived PDXs (average of 43.7; ranging from 13.26 to 105.93) compared to RHGP PDXs (average of 8.55; ranging from 0.007 to 47.07) (Supplementary Fig. 9a, b). As observed for Claudin-2, mixed metastases demonstrated an intermediate Claudin-8/Tubulin ratio (Supplementary Fig. 9b). An assessment of the mixed lesions revealed that the Claudin-8/Tubulin ratio was positively correlated with lesions that have a predominant contribution of DHGP features (Supplementary Fig. 9c; Supplementary Table 2). These data demonstrated that, as observed in patient-derived samples CRCLMs, Claudin-8 is expressed at higher levels in subcutaneous PDX tumors derived from DHGP-type liver metastases whereas Claudin-2 is expressed at higher levels in subcutaneous PDX tumors derived from CRC patients with RHGP-type liver metastases.

### DHGP PDX models express Claudin-8 while RHGP PDX models express Claudin-2

**Fig. 5:**
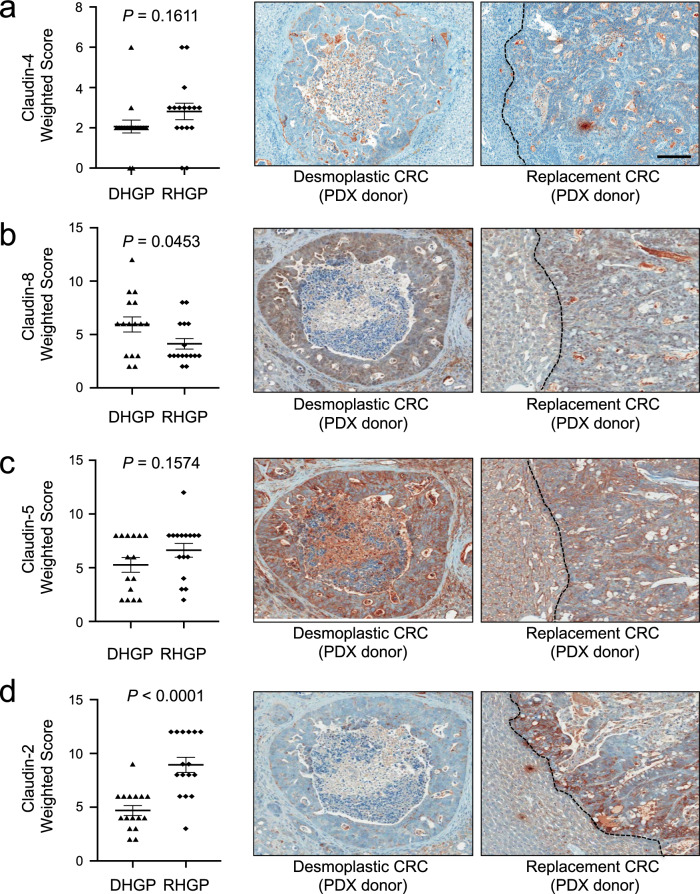
Claudin-2 is enriched in the PDXs derived from replacement-type liver metastases. **a**–**d** Paraffin embedded sections from established intrahepatic PDXs from either DHGP- or RHGP-type lesions were subjected to immunohistochemical staining with anti-Claudin-4 (**a**), anti-Claudin-8 (**b**), anti-Claudin-5 (**c**), or anti-Claudin-2 (**d**) antibodies. Claudin-8 expression is significantly enriched in the desmoplastic-type liver metastases. In contrast, Claudin-2 expression is significantly increased in the replacement-type liver metastases. Representative images of Claudin-4, Claudin-8, Claudin-5, or Claudin-2 staining are shown. Scale bar = 100 μm and applies to all panels. Scoring of IHC staining was performed by two independent pathologists (AO and AG). Data are presented as the mean ± SE.

In human specimens, Claudin-2 is enriched in RHGP CRCLMs, Claudin-8 is expressed in DHGP CRCLMs and Claudin-4 is detected in both types of metastases (Fig. 3, Supplementary Fig. 7). We next assessed if these expression patterns were maintained in our established PDX models of RHGP or DHGP CRCLMs following intrahepatic implantation. IHC staining of PDX-derived liver metastases revealed that Claudin-4 was expressed equally in both RHGPs and DHGPs (Fig. 5a). In agreement with the results obtained from clinical samples, significant enrichment of Claudin-8 expression in DHGP liver metastases was observed (Fig. 5b). A nonstatistically significant trend for an increase in Claudin-5 expression in RHGP-type lesions was observed (Fig. 5c). Finally, Claudin-2 was more highly expressed in the RHGP-type metastatic lesions relative to DHGP-type lesions (Fig. 5d). Together, these observations revealed that PDX-derived liver metastases recapitulated the Claudin expression pattern observed in clinical samples from patients with CRCLMs.

### Claudin-2 is present in circulating extracellular vesicles from CRC patients with RHGP liver metastases

**Fig. 6:**
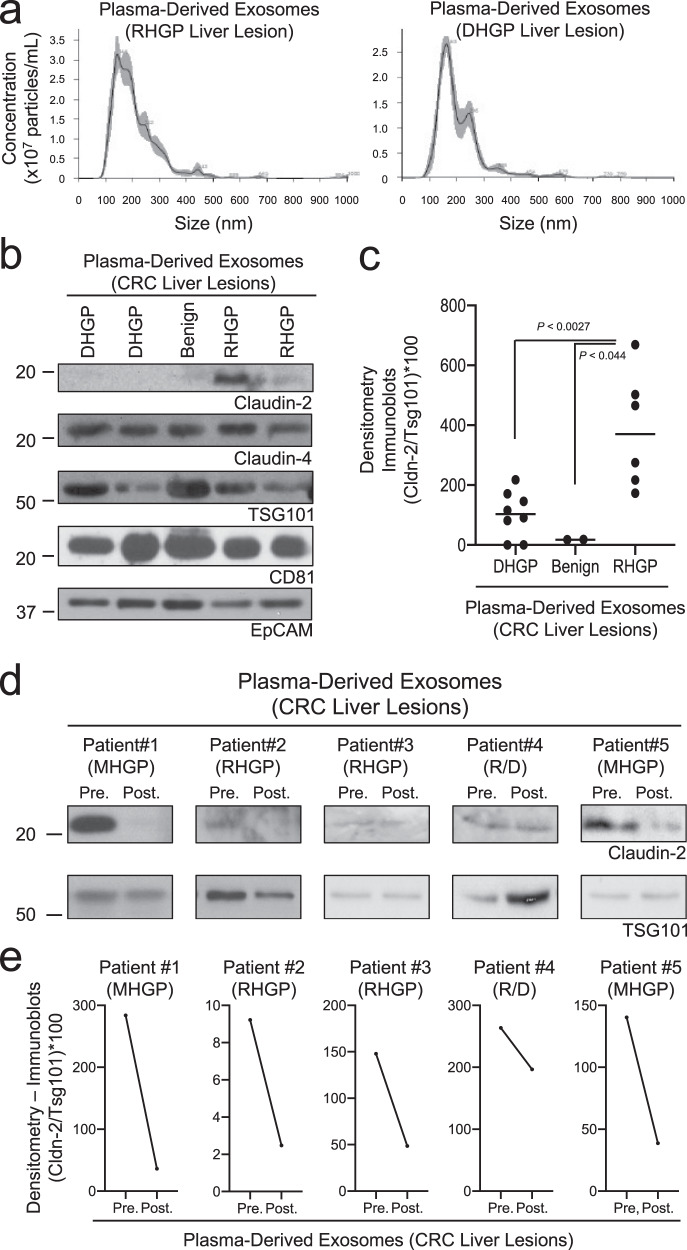
Claudin-2 is enriched in extracellular vesicles isolated from patients with replacement-type colorectal cancer liver metastases. **a** Nanoparticle tracking analysis of EVs detected in plasma derived from either a RHGP liver lesion bearing patient or a DHGP liver lesion bearing patient. **b** Representative immunoblot analysis of Claudin-2, Claudin-4, CD81, EpCAM, and TSG101 using lysates prepared from EVs isolated from patients with the indicated lesions. **c** Claudin-2 levels in EVs concentrated from plasma of liver-metastatic CRC patients with the indicated lesions were normalized to TSG101 levels. **d** Immunoblot analysis of Claudin-2 and TSG101 using lysates prepared from EVs concentrated from patients, pre- and post-resection, with the indicated lesions. **e** Claudin-2 levels in EVs concentrated from plasma, pre- and post-resection, of liver-metastatic CRC patients with the indicated lesions were normalized to TSG101 levels.

Currently, the only way CRCLMs can be segregated into distinct histopathological growth patterns is through the evaluation of H&E stains of resected liver metastases. Due to the possible heterogeneity of the HGPs within a single lesion, biopsy material is not sufficient to categorize these metastases^2^. Given that CRCLMs with DHGP features respond more favorably to chemotherapy plus antiangiogenic therapy when compared to RHGP-type liver metastases^8^, a noninvasive method to determine the type of HGP would be advantageous to select optimal treatment regimens for patients with CRCLM. Emerging data indicate that Claudins present in extracellular vesicles (EVs), including microvesicles and exosomes, may be useful biomarkers in diverse cancers^44–47^. Thus, we next explored the possibility that release of claudin-containing EVs could potentially stratify CRC patients with liver metastases based on HGP. Nanoparticle tracking analysis (NTA) revealed that circulating EVs isolated from the blood of CRC liver metastasis patients, regardless of the histopathological growth pattern, showed a maximal EV peak between 150 and 200 nm (Fig. 6a). We determined whether Claudins expressed in both types of metastases (Claudin-4), or those differentially expressed between DHGP (Claudin-8) and RHGP (Claudin-5, Claudin-2) lesions, could be detected in EVs concentrated from blood samples obtained from CRCLM patients (Supplementary Table 3). EVs were enriched from the plasma by ultracentrifugation and assayed for the presence of EpCAM, Claudin-4, Claudin-8, Claudin-5, and Claudin-2 by immunoblot analysis. In addition, TSG101 and CD81 were included as EV markers. Both Claudin-4 and Claudin-2 were readily detectable; however, we were unable to detect signals for Claudin-5 or Claudin-8 (Fig. 6b).

Our previous data suggested that Claudin-2 may serve as a useful marker to identify RHGP-type liver metastases (Figs. 2–5); thus, we next assessed whether an increase in circulating Claudin-2-positive EVs could identify patients with RHGP-type CRCLMs. Plasma samples were collected from eight chemo-naïve patients with DHGP-type liver metastases, six chemo-naïve patients with RHGP-type metastases and two patients with benign lesions (cyst) (Supplementary Fig. 10a, Supplementary Table 3). As anticipated, Claudin-2 levels appear elevated only in EVs derived from patients with RHGP-type liver metastases (Fig. 6b). The ratio of Claudin-2 to TSG101 was determined to control for EV content in each sample. These analyses revealed that EVs derived from patients with RHGP-type CRCLMs exhibited higher expression of Claudin-2 relative to EVs isolated from patients with DHGP-type CRCLMs (Fig. 6c). No cancer-derived Claudin-2 was detected in EVs isolated from patients possessing benign lesions (Fig. 6c). To further assess the predictive potential of Claudin-2 to identify RHGP lesions, we reasoned that Claudin-2 levels in circulating EVs would decrease following liver metastasis resection. Thus, plasma samples were collected from five patients pre-(in the operating room just prior to surgery) and post-resection (2–4 weeks after surgery; all patients were disease free) (Supplementary Fig. 10b, Supplementary Table 3). Claudin-2 levels were found to be decreased following resection in all patients (Fig. 6d-e). Thus, high levels of Claudin-2 in EVs isolated in the blood circulation of CRC patients with liver metastases may predict the likelihood that the metastases possess features of a RHGP-type lesion.

## Discussion

In the current study, we implicate Claudin-2 as a critical determinant in the formation of RHGP-type CRC liver metastases. Claudin-2 promotes anchorage-independent growth of colorectal cancer cells and augments their survival within the blood circulation and during early seeding events by conferring anoikis resistance^30,36,48^. We now demonstrate that Claudin-2 promotes the formation of CRC liver metastases. These data are in agreement with previous results showing that Claudin-2 mediates breast cancer liver metastasis, in part, by providing an early survival advantage after seeding the liver^24,25,27,38^. Thus, colorectal cancer cells may also form replacement-type liver metastases through interactions with resident hepatocytes, which rely on the formation of *trans*-homotypic Claudin-2–Claudin-2 interactions between these cell types. Our previous work implicated the first extracellular loop of Claudin-2 as an essential determinant for tumor cell-hepatocyte interactions and liver metastasis^27^. Our current results indicate that such cancer cell/hepatocyte interactions do not rely on the PDZ-binding motif of Claudin-2. However, the PDZ-binding motif found in Claudins is important for their ability to promote metastasis^25,49^, and we demonstrate that the PDZ-binding motif of Claudin-2 is required for CRC liver metastasis. Thus, it is conceivable that the Claudin-2 PDZ-binding motif confers early cancer cell survival within the liver. Although potential interacting proteins that bind this motif remain poorly defined, recent studies have identified Pdlim7 and Afadin as Claudin-2 interacting partner, which contribute to the ability of breast cancer cells to grow in soft agar and metastasize to the lungs and/or liver^25,50^. Similar signaling pathways downstream of Claudin-2-Afadin may be involved in both breast and CRC liver metastasis. The precise molecular mechanisms through which Claudin-2 and Afadin contributes to the metastatic phenotype requires further experimentation.

Claudin-2 is overexpressed in human CRC cells, which is associated with enhanced cell proliferation, colony formation or self-renewal in vitro^30,32,34,36^. Furthermore, Claudin-2 overexpression in late-stage CRC is associated with poor post-treatment disease-free survival^34^. In agreement with these findings, we demonstrate that elevated Claudin-2 expression in primary CRCs is associated with a significant decrease in overall survival as well as a trend for reduced relapse-free survival. However, we now also demonstrate that elevated Claudin-2 levels in CRC primary tumors is significantly correlated with an increased risk of developing liver metastases within 5 years. Our data suggest that Claudin-2 levels start and remain high throughout the metastatic process, which facilitates the establishment of CRC cells in the liver. This differs from our observations in breast cancer, where Claudin-2 is weakly expressed in primary breast cancers cells but is highly expressed in breast cancer liver metastases^24,27^.

CRC cells can establish liver metastasis that are classified into three predominant subtypes, which include pushing (PHGP)-, desmoplastic (DHGP)-, and replacement (RHGP)-type lesions^2,3,8,9^. These HGPs are associated with different outcomes. Indeed, patients with CRCLMs that are categorized as the DHGP-type lesions have significantly better overall survival rates when compared with patients that possess CRCLMs that are characterized as RHGP- or PHGP-type liver metastases^2^. While the HGPs can now be accurately scored according to the proposed guidelines following evaluation of H&E stained sections^2^, specific markers associated with each histopathological growth pattern are beginning to emerge. RNA-Seq analyses revealed distinct expression patterns for specific Claudin family members in DHGP- and RHGP-type liver metastases. IHC validation suggests that differential immunohistochemical staining with Claudin-2 and -8 could help define CRCLM HGPs. Our results suggest that CRCLMs belonging to the RHGP will display a Claudin-2^high^/Claudin-8^low^ expression pattern compared to DHGP CRCLMs, which would possess a Claudin-2^low^/Claudin-8^high^ profile. This observation is in agreement with the fact that Claudin-2 promotes breast cancer liver metastasis, which more often form RHGP-type lesions^8,9,24,27,51^. While our RNA-Seq data reveal that several Claudin family members are expressed in CRC liver metastases with replacement-type features, we suggest that Claudin-2 represents a superior biomarker for RHGP CRCLMs due to the fact that it was the only claudin family member that was upregulated in breast cancer liver metastases and functionally shown to promote both breast and CRC metastasis to the liver^24,26,27,32,35^. In contrast, Claudin-8 was the only family member whose expression was detected only in DHGP CRCLMs.

Claudin-2 expression is inversely correlated with molecular subtypes association with increased angiogenesis (e.g., CMS4), a feature of the DHGP-type liver metastases. In contrast, Claudin-2 expression was positively correlated with molecular subtypes characterized by an inflammatory signal (CMS1, CRIS-A), metabolic dysregulation (CMS3, CRIS-A), microsatellite instability (MSI, CMS1, CRIS-A), or PI3K and MAPK pathway mutations (CMS3, CRIS-A, CRIS-E)^52^. Claudin-2 expression is also more highly correlated with the CMS1 subtype, which is associated with the worse survival after relapse^42^. In agreement with our findings, upregulation of glycolytic genes and enhanced glycolytic capacity was detected in metastatic colorectal cancer cells, which was associated with metastasis and poor survival in colorectal cancer patients^53,54^. Interestingly, KRAS mutation was highly correlated with Claudin-2 expression in CRCLMs in our study. It has been suggested previously that KRAS mutations are associated with more aggressive metastatic behavior of CRCLMs, worse overall survival after CRCLM resection and the RHGP^55–57^. This is in agreement with the central role that the ERK signaling pathway plays in regulating Claudin-2 expression in a variety of cancer cell types^35,38,58–62^.

Currently, patterning of CRCLMs can only be accomplished by histopathological evaluation of resected samples from patients with liver metastases^2^. Given the importance of defining the histopathological growth pattern for patient prognosis and for defining optimal treatment regimens^8^, there is significant interest in developing noninvasive biomarkers to identify patients with RHGP or DHGP-type CRCLMs. Primary tumors and metastases produce and release EVs into the bloodstream, which can be used for cancer diagnosis or monitoring disease progression^63^. Interestingly, claudins in EVs are emerging as circulating biomarkers in a variety of cancer types^44–47^. Our findings suggested that plasma from CRC patients with RHGP-type liver metastases have Claudin-2-containing EVs in circulation that could be exploited for HGP segregation or disease monitoring following treatment. These approaches could be extended to other solid cancers that spread to the liver and form distinct types of metastases (DHGP, RHGP); including gastric cancer, pancreatic cancer, uveal melanoma, or cutaneous melanoma^64–67^.

While molecular insights underlying the differences between DHGP- and RHGP-type lesions are beginning to emerge, progress has been hampered by a lack of robust models that accurately represent DHGP-type CRCLMs. Indeed, spontaneous or experimental liver metastasis assays using CRC cell lines only give rise to RHGP-type lesions^8^. Interestingly, knockdown of Arp2/3, a gene mediating cancer cell motility, was able to revert HT-29-derived liver metastases from a RHGP to a DHGP-type lesion, representing one of the few existing models of DHGP CRCLMs^8^. To address this deficiency, we have successfully established PDXs models that represent both the DHGP- and RHGP-type of CRCLMs. Using these novel PDX models we have been able to validate the expression of Claudin-2 as a marker for the RHGP type of CRCLMs. These models will also be useful for preclinical drug testing providing a unique opportunity to test non-conventional therapies and novel drug combinations to better treat RHGP CRCLMs. They will also represent useful tools to mechanistically define molecular and cellular mechanisms that contribute to the formation of DHGP and RHGP CRCLMs.

Together, our results suggest that Claudin-2 may represent a useful marker and a rationale therapeutic target for CRCLMs of the RHGP subtype. Antibody-drug conjugates targeting Claudin-2 may represent a viable therapeutic strategy to better manage CRC patients with RHGP-type liver metastases. In this regard, Claudin-4 targeting has been demonstrated to enhance the antitumor effects of chemotherapy or other targeted therapeutic agents^68^. Furthermore, a human-rat chimeric monoclonal antibody that recognizes the extracellular domains of human and mouse Claudin-2 has been generated and proven to be safely distributed into the liver, kidney, and tumor tissues of mice bearing Claudin-2-expressing fibrosarcoma cells^69^. Such anti-Claudin-2 targeting reagents could be useful in the management of liver metastases from multiple solid cancers that exhibit replacement-type features.

## Methods

### Cell culture and transfections

The HT-29 and SW403 cell lines were obtained from the American Type Culture Collection (ATCC). Claudin-2 deficient HT-29 cells were engineered using a CRISPR/Cas9 approach as described previously^25^. The precise nature of the CRISPR/Cas9 mediated mutagenesis was sequence verified to ensure that each clone within the reconstituted pooled population (Claudin-2^KO^) carried the expected gene disruptions. Exogenous Claudin-2 expression was achieved by cloning Claudin-2 cDNA into pQXCIB (Clontech) expression vector. The HA-tagged versions of Claudin-2 were constructed by inserting a tandem HA-tag in the intracellular loop. Virus production and cell infection was performed as previously described^27^. Cells were selected and maintained in 2 μg/mL of puromycin (Cat. Code: ant-pr, Invivogen) and/or 7.5 mg/mL Blasticidin (Cat. Code: ant-bl, Invivogen).

### Experimental metastasis assays

For experimental liver metastasis assays, HT-29 or SW403 cells (1 × 10^6^) were injected into the spleens of 4- to 6-week-old female Scid/beige mice as previously described^24^. Experimental lung metastasis assays were performed by injecting 5 × 10^5^ cells into the tail veins of 4- to 6-week-old female Scid/beige mice as previously described^25^. For spontaneous metastasis assays, 1 × 10^6^ cells were injected in the caecum and tumor wet weight determined at the time of autopsy. Tumor burden in the liver or lungs was quantified using Imagescope software (Aperio) as previously described^24^.

Claudin-2-deficient C57BL/6 J mice (kindly provided by Dr. Alan Yu)^70^ were back-crossed for 10 generations to yield Claudin-2-deficient mice on a Balb/c background. The mice were housed in facilities managed by the McGill University Animal Resources Centre and all animal experiments were conducted under a McGill University approved Animal Use Protocol in accordance with guidelines established by the Canadian Council on Animal Care.

### Hepatocyte adhesion assays

To assess the ability of HT-29-derived colorectal cancer cells to adhere to hepatocytes, 1 × 10^5^ carboxy-fluorescein diacetate, succinimidyl ester (CFDA SE; Invitrogen)-labeled cancer cells were seeded onto a monolayer of primary hepatocytes from either wild-type or Claudin-2-deficient mice as previously described^27,70^. After a 1 h incubation period at 37 °C, plates were washed twice in phosphate-buffered saline (PBS) then fixed for 20 min. in 10% formalin (Fisher Scientific). After two washes in PBS, cancer cells that remained adhered to the hepatocyte monolayer were visualized using epifluorescence. For each experiment, data are expressed as the number of fluorescent cells per field. Data represent the averages from at least three independent experiments (five fields/well and four wells/experiment).

### Immunoblotting

Cell or tumor lysates were generated, total proteins were separated by electrophoresis, transferred to membranes and processed as previously described^24^. Immunoblot analyses were performed using the following antibodies: Claudin-2 (1:5,000; Cat. #: 325600, Thermofisher Scientific), Claudin-4 (1:5,000; Cat. #: 329400, Thermofisher Scientific), CD81 (1:2,000; Cat. #: 10630D, Thermofisher Scientific), EpCAM (1:2,500; cat. #: 93790, Cell Signaling Technology), TSG101 (1:2,000; Cat. #: ab83, abcam), α-Tubulin (1:10,000; Cat. #: T9026, Sigma), and HA.11 clone 16B12 (1:10,000; Cat. #: MMS-101-P-200, Covance) antibodies. Membranes were incubated with their corresponding Horseradish-peroxidase-conjugated anti-IgG secondary antibodies (Jackson ImmunoResearch Laboratories, Inc.) and visualized with chemiluminescent HRP Substrate (Cat. #: WBLUF0500, Millipore) or an enhanced chemiluminescence system (Cat. #: 34578, Thermofisher).

### Subcutaneous or intrahepatic implantation of tumor fragments

Human tumor fragment transplantation was performed as previously described^71^. Briefly, fresh resected CRC liver metastasis or biopsy samples were collected from the operating theatre and held on ice in DMEM/F12 medium supplemented with Penicillin/Streptomycin, Gentamycin and Amphotericin B. The sample was then divided into ~1 mm^3^ fragments. For subcutaneous implantation, a 2–3 mm skin incision was made on the flank of the animal and a subcutaneous pocket created, into which tumor fragments were inserted after being dipped in Matrigel (Cat. #: 354234, BD Biosciences). The skin incision is then closed with wound clips. Tumor growth was monitored on a weekly basis. For intrahepatic implantation, the cardiac lobe of the liver was first exposed, and a cotton-tipped applicator placed underneath to stabilize the exposed tissue. A 2 mm long × 2 mm deep incision was made on the surface of the liver and a piece of Surgicel applied to the incision site to achieve hemostasis before inserting a tumor fragment. A small piece of Surgicel was then placed over the liver incision to maintain the fragment in place and ensure hemostasis. The peritoneum was then closed with sutures and the skin closed with wound clips. Following intrahepatic implantation, liver metastases were left to grow in mice for up to 5 months before sample collection.

### RNA-seq data analysis

To generate the *Claudin* expression pattern in DHGP and RHGP samples, we complied gene expression from GSE151165^39^. Claudin differential gene expression analysis was performed using DESeq2 (version 1.16.1) Bioconductor R package^72^. The principal component analysis (PCA) was performed with the prcomp() R function and visualized with the pca3d (version 0.10) CRAN R package. Heatmaps were generated with heatmap.2 from gplots (version 3.0.1) R package.

### Gene expression analysis

Gene expression (RSEM values) and mutation data for 18,939 genes from 377 colon adenocarcinoma samples (TCGA PanCancer Atlas)^40^ were downloaded from the cBioPortal for cancer genomics (https://www.cbioportal.org) using the R-package *cgdsr* (v1.3.0). Molecular subtypes (i.e., CMS, CRIS, and MSI) were determined using the BioConductor package *CMScaller* (v0.99.2) based on the log2-transformed RSEM values. Classifications were only considered when the false discovery rate was inferior to 10%. *CLDN2* expression was compared between subtypes using nonparametrical Wilcoxon signed rank test or Kruskal-Wallis test were appropriate (i.e., 2-group and multi-group comparisons, respectively). Expression differences for *CLDN2* between colon adenocarcinoma samples stratified by mutation status for the 25 most recurrently mutated genes in the present series were evaluated in a similar fashion. All statistical analyses and visualizations were performed in R using base functions and the *ggplot2* package (v3.3.1), respectively. *P*-values inferior to 5% were considered significant.

### Immunohistochemistry

Immunohistochemical staining was performed using mouse monoclonal antibodies against Claudin-2 (1:250; clone 12H12, Thermofisher Scientific) and Claudin-4 (1:100; Cat. #: 364800, Thermofisher Scientific). Rabbit polyclonal antibodies against Claudin-5 (1:300; Cat. #: 341600, Thermofisher Scientific), Claudin-8 (1:50; Cat. #: 400700Z, Thermofisher Scientific), and CK20 (1:200; Cat. #: M7019, Dako) were also used. Serial sections were stained using a Discovery Ultra autostainer (Ventana Medical System Inc.) following manufacturer’s instructions. Each sample was given a semi-quantitative score from 0 to 4 for the proportion of tumor cells staining positive [1 (**<**25%), 2 (<25%; **>**50%), 3 (<50%; **>**75%), and 4 (**>**75%)] and 0–3 for the intensity of tumor cell staining [0 (no staining), 1 (weak), 2 (moderate), and 3 (strong)]. The proportion and intensity scores were combined by multiplication to obtain a final weighted-score ranging from 0 to 12. Two independent pathologists (AO, GA) performed the scoring.

### Tissue sample acquisition

Informed consent was obtained from all patients through the MUHC Liver Disease Biobank (LDB: MUHC research ethics board approved protocol SDR-11-066). Surgical specimens were procured and released to the Biobank immediately after the pathologist’s confirmation of carcinoma and surgical margins. Biological materials were also provided by the Ontario Tumour Bank (OTB), which is funded by the Ontario Institute for Cancer Research (OICR).

### Clinical data

This study included a total of 97 CRC primary tumors (47 from the LDB and 50 from OTB). Matched resected liver-metastatic lesions from 22 chemo-naïve patients, representing 23 chemo-naive lesions (8 DHGP and 15 RHGP), were also obtained from the LBD.

In addition, FFPE samples of 64 CRCLM resection specimens were obtained from the biobank of the GZA Hospital Sint-Augustinus, Antwerp, Belgium (Federal Agency Notification Number BB190028 obtained after approval by the local ethics review board). The samples were entered into the biobank after written informed consent of patients in the Sint-Augustinus Hospital, Antwerp, Belgium (*n* = 39), and in the Erasmus MC Cancer Institute, Rotterdam, The Netherlands (*n* = 25). Standard perioperative systemic treatment was given in 31 patients.

Histopathological scoring of the growth patterns was performed using the consensus guidelines^2^ by experienced pathologists (Z-HG (McGill cohort) and PV (European cohort)).

### Extracellular vesicle concentration from patient plasma

For EV concentration, 10–20 mL of whole blood was collected in EDTA-coated tubes from patients just prior to resection. Whole blood in EDTA tubes was kept at room temp until plasma separation. For each patient, 4 mL of plasma was then snap frozen in cryovials placed in liquid nitrogen and stored in −80 °C freezer. To concentrate EVs, frozen tubes were thawed at room temperature. Concentration of EVs by differential- and ultracentrifugation was performed as described previously, with minor modifications^73^. Briefly, normal human blood plasma (5 mL) was diluted with 30 mL PBS and differentially centrifuged at 4700 × *g* for 15 min at 4 °C to eliminate cell debris, followed by high-speed centrifuge at 10,000 rpm for 25 min at 4 °C. The supernatant was gently collected with a serological pipette, loaded into a syringe and filtered using a 0.22 μm filter (Ultident Scientific; Cat#229747). Samples were ultracentrifuged at 110,000 × *g* for 70 min at 4 °C, the supernatant discarded, and the pellet resuspended in PBS up to 20 mL. Samples were subjected to further ultracentrifugation at 110,000 × *g* for 70 min at 4 °C to pellet EVs. The resulting pellet was resuspended in 150 μl PBS, aliquoted and kept at −20 °C without any additives used. The size and concentration of nanoparticles in EV preparations was measured using a NanoSight NS500 (NanoSight) instrument. All samples produced readily detectable EVs (Fig. 6a), with the majority of the EV population within the size range associated with exosomes (150–200 nm)^74^. Samples were then stored at −80 °C until analyzed by immunoblotting for the expression of TSG101 and CD81.

### Statistics and reproducibility

Unless specified, all significance values were calculated using a Student’s *t*-test. Significance values associated with claudin expression status in subcutaneous tumors (Fig. 4 and Supplementary Fig. 9) were calculated using a Mann–Whitney test. CRC overall survival and relapse-free survival (Supplementary Fig. 6d and e) curves were plotted using the Kaplan–Meier estimator and the log-rank test was used to evaluate significant differences. The statistical software package Prism (Graphpad) was used.

### Reporting summary

Further information on research design is available in the Nature Research Reporting Summary linked to this article.

## Supplementary information

Supplementary Information

Description of Supplementary Files

Supplementary Data 1

Reporting Summary

## Data Availability

RNA-seq data have been made available publicly in GEO (GSE151165):686 Source data are available in Supplementary Data 1. All other data are available from the corresponding author on reasonable request.
