# Supplementary material for: Claudin-2 promotes colorectal cancer liver metastasis and is a biomarker of the replacement type growth pattern
Source: Commun Biol. 2021 Jun 2;4:657. doi: 10.1038/s42003-021-02189-9 (PMC8172859; doi:10.1038/s42003-021-02189-9)
Supplement: Supplementary file 1 — Supplementary Information [file 42003_2021_2189_MOESM1_ESM.pdf]

Claudin-2 IHC: Primary CRC Tumors (HT-29)

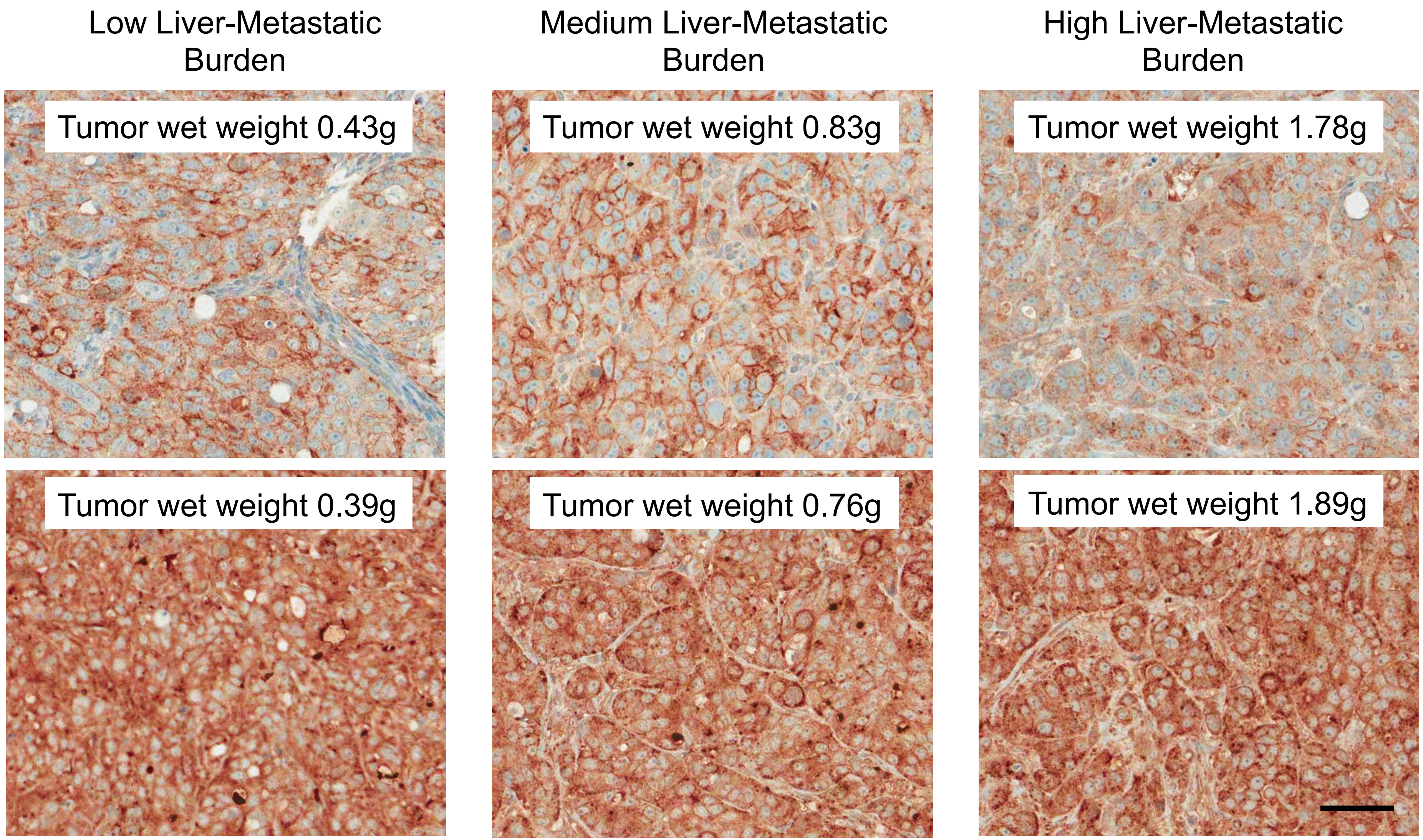

Tabariès *et al.*, Supplementary Figure 1

Supplementary Fig. 1: Claudin-2 expression in HT-29-derived primary CRC tumors is not correlated with the degree of spontaneous liver metastasis. Representative images of Claudin-2 IHC performed on primary CRC tumors (intra-caecal injection) shown in Figure 1d of the manuscript. Scale bar = 50 mm and applies to all panels.

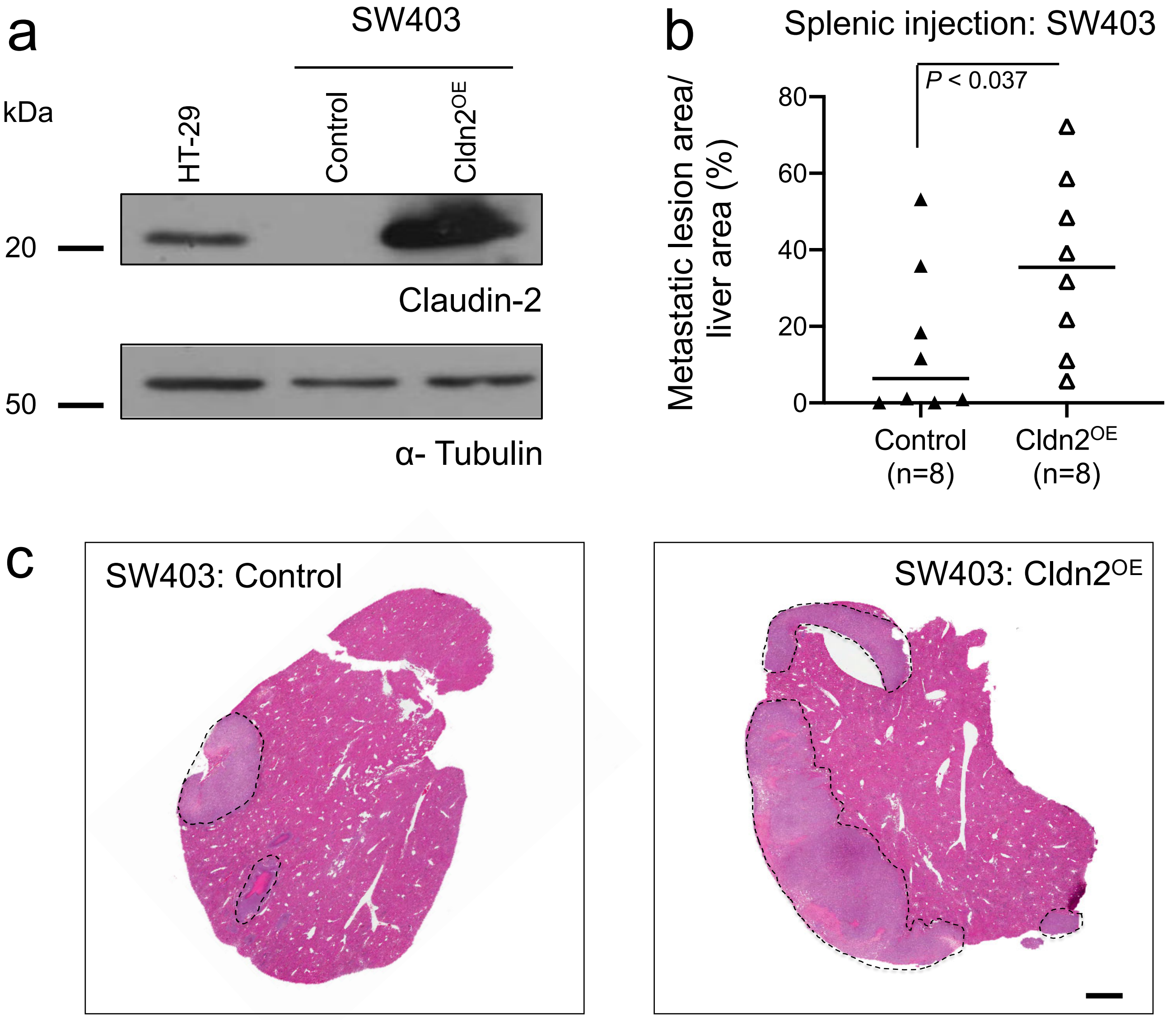

Tabariès *et al.*, Supplementary Figure 2

Supplementary Fig. 2: Claudin-2 expression promotes efficient colorectal cancer liver metastasis. a Immunoblot analysis of Claudin-2 expression in parental SW403 colorectal cancer cells (Ctrl) or SW403 cells engineered to express exogenous claudin-2 ($cldn2^{OE}$). A whole cell lysate from HT-29 cells serves as a positive control. As a loading control, whole cell lysates were blotted for α-Tubulin. **b** Quantification of the metastatic burden (tumor area/tissue area) within the cardiac liver lobe following splenic injection. **c** Representative H&E images of the cardiac liver lobe are shown for mice injected with the indicated cell populations. Dotted lines circumscribe colorectal cancer metastatic lesions within the liver. Scale bar = 2 mm and applies to both panels.

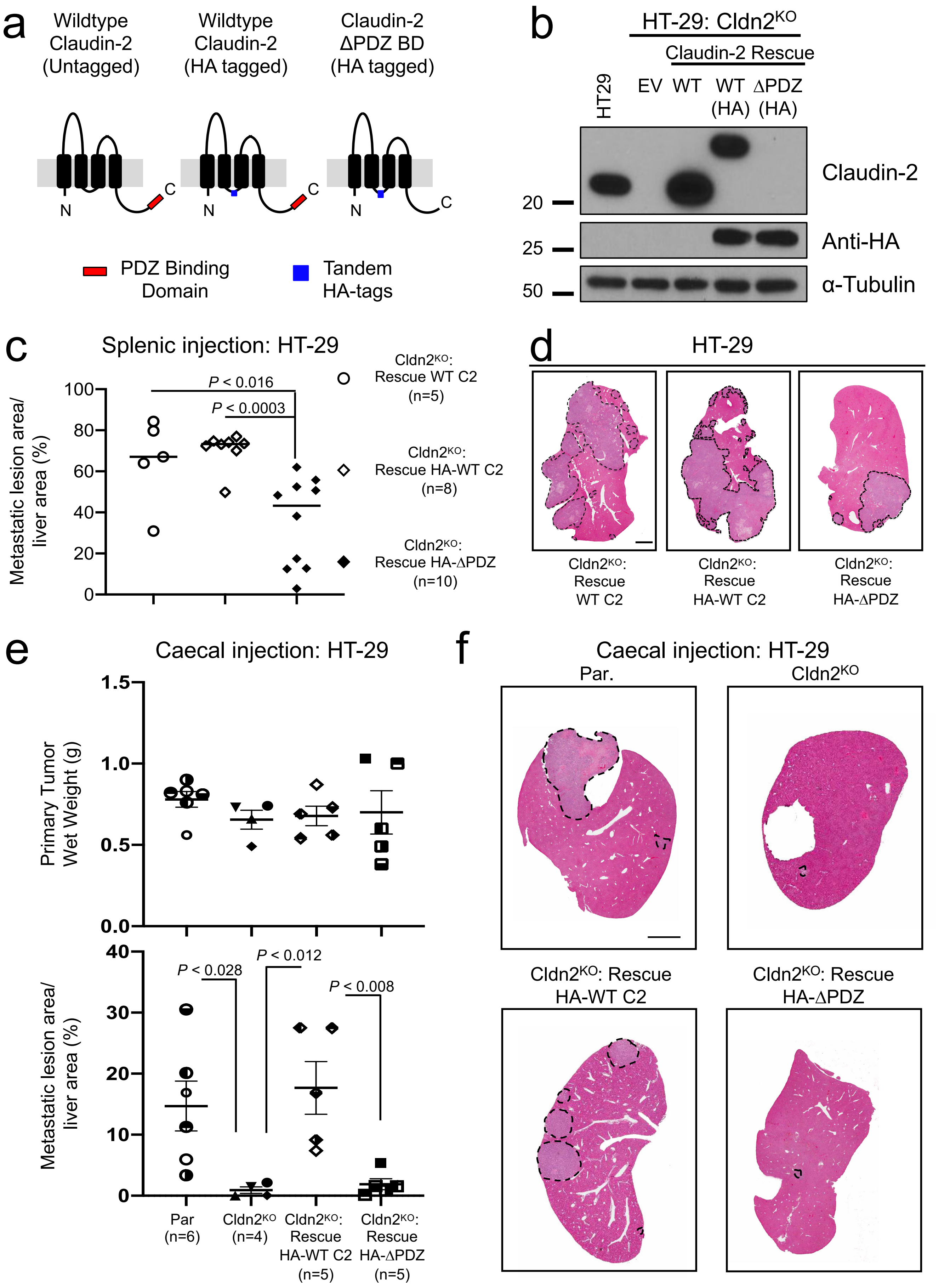

Tabariès *et al.*, Supplementary Figure 3

Supplementary Fig. 3: The Claudin-2 PDZ-binding motif contributes to efficient colorectal cancer liver metastasis. a Schematic of Claudin-2 indicating the presence of the H-Influenza hemagglutinin (HA) tag in the cytoplasmic loop of wild-type and ΔPDZ BD Claudin-2 mutant. **b** Claudin-2 expression in the indicated HT-29 derived cell populations was analyzed by immunoblotting with anti-Claudin-2 and anti-HA antibodies. α-Tubulin served as a loading control. **c** Liver-metastatic burden (lesion area/tissue area) was analyzed following splenic injection of the indicated cell lines. **d** Representative H&E images of the cardiac liver lobe from mice injected with the indicated cell populations are shown. Scale bar represents 2 mm and applies to all panels. **e** Quantification of the primary tumor burden (wet weight) and metastatic burden (lesion area/tissue area) within the cardiac liver lobe following caecal injection. **f** Representative H&E images of the cardiac liver lobe are shown for mice injected (caecal) with the indicated cell populations. Scale bar, 2 mm and applies to all panels. Data are presented as the mean ± SE.

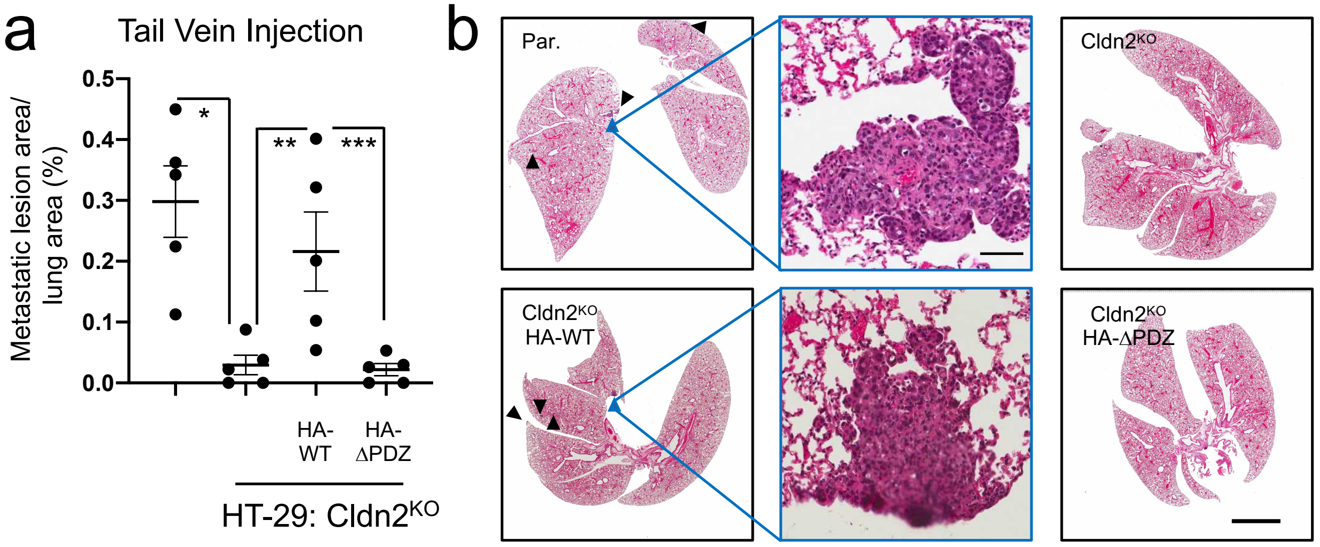

Tabariès *et al.*, Supplementary Figure 4

Supplementary Fig. 4: Claudin-2 mediates efficient colorectal cancer lung metastasis. **a** Lung-metastatic burden (tumor area/tissue area) was analyzed following tail vein injection of the indicated cell lines (*, P = 0.0022; **, P = 0.024; ***, P = 0.0185). **b** Representative images of the lungs for each cell population are shown. Scale bar represents 2 mm and applies to all panels. Scale bar within the inset represents 50 μm and applies to all panels. Data are presented as the mean ± SE.

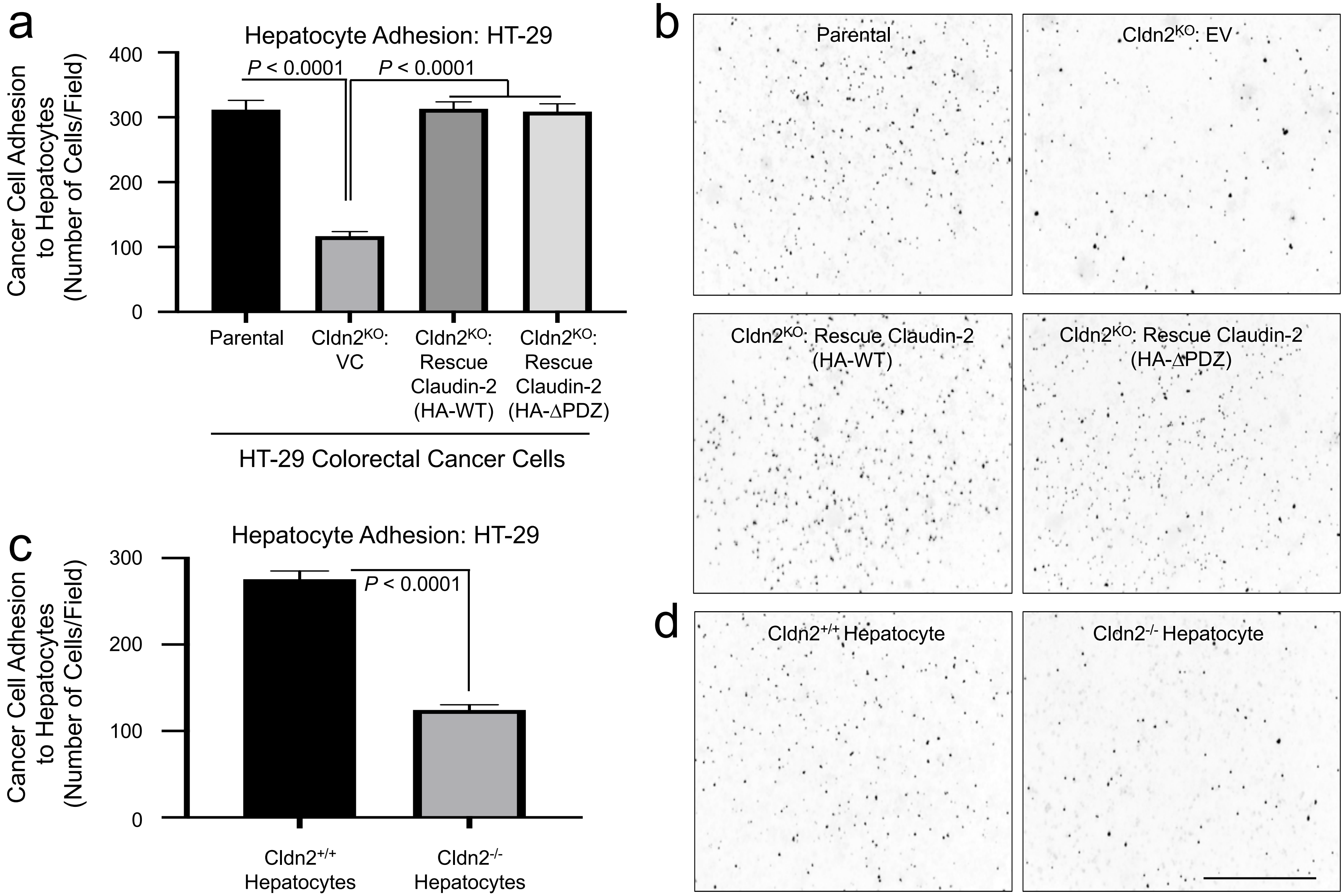

Tabariès *et al.*, Supplementary Figure 5

Supplementary Fig. 5: Claudin-2 functions to promote colorectal cancer cell adhesion to hepatocytes. **a** The indicated colorectal cancer cells were plated onto primary hepatocyte monolayers and adhesion was quantified after 1 h. Claudin-2 deficiency in HT-29 cells resulted in statistically significant decreases in hepatocyte adhesion compared to parental cells. The phenotype was rescued upon expression of either the wild-type Claudin-2 or the ΔPDZ BD Claudin-2 mutant. **b** Representative images of each cancer cell population following adhesion to primary hepatocyte monolayers are shown. **c** Human HT-29 colorectal cancer cells (expressing Claudin-2) were analyzed for their abilities to adhere to either Claudin-2 proficient or deficient primary hepatocyte monolayers. Loss of Claudin-2 expression in primary hepatocytes resulted in statistically significant decreases in hepatocyte adhesion compared to control hepatocytes. **d** Representative images are shown following cancer cell adhesion to a monolayer of primary hepatocytes. The scale bar (right) represents 200 μm and apply to all panels in **b** and **d**. VC: vector control. Data are presented as the mean ± SE.

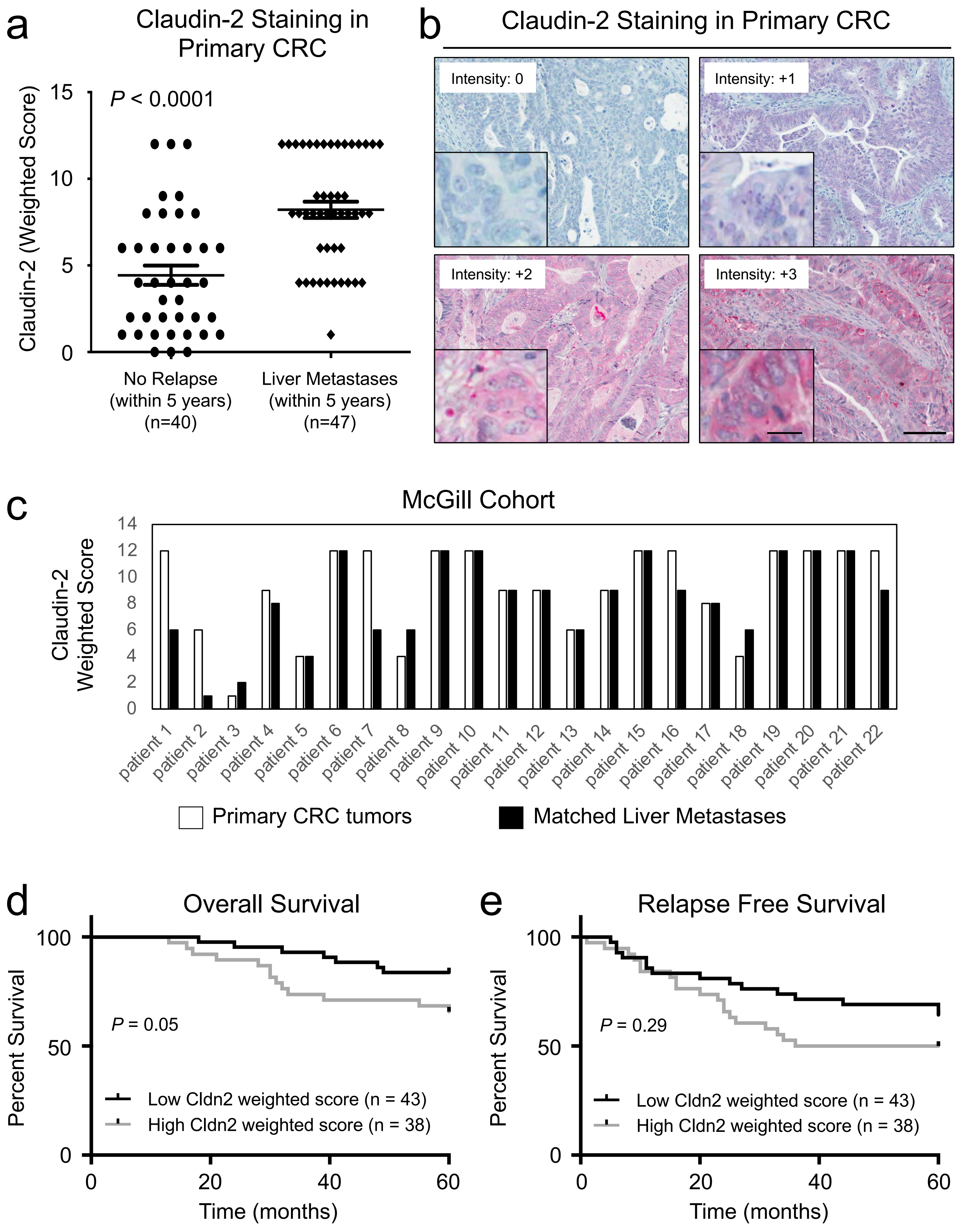

Tabariès *et al.*, Supplementary Figure 6

Supplementary Fig. 6: High Claudin-2 expression in primary colorectal cancers is associated with the rapid formation of liver metastases. a Quantification of Claudin-2 immunohistochemical staining of paraffin embedded sections from primary colorectal cancers (CRC). A total of 40 primary CRC tumors from patients with no known liver-specific relapse within 5 years and 47 primary CRC samples patients with relapse to the liver within 5 years were analyzed. Scoring of Claudin-2 staining (percentage positivity and intensity) was performed by two independent pathologists (AO, GA). **b** Representative images of Claudin-2 IHC from each category (0, +1, +2, +3) are shown. Scale bar = 100 μm and applies to all panels. Scale bar in inset =20 μm and applies to all panels **c** Paraffin embedded sections from primary colorectal cancers and their matched liver metastases from 22 patients were subjected to immunohistochemical staining with anti-Claudin-2 antibodies. A weighted score for Claudin-2 staining (percentage positivity and intensity) in each sample was provided by two independent pathologists (AO and GA). **d** High Claudin-2 weighted score (equal or higher than 8) in primary tumors from colorectal cancer patients is significantly associated with poor overall survival. **e** High Claudin-2 weighted score in primary tumors from colorectal cancer patients is associated with poor relapse-free survival. Data are presented as the mean ± SE.

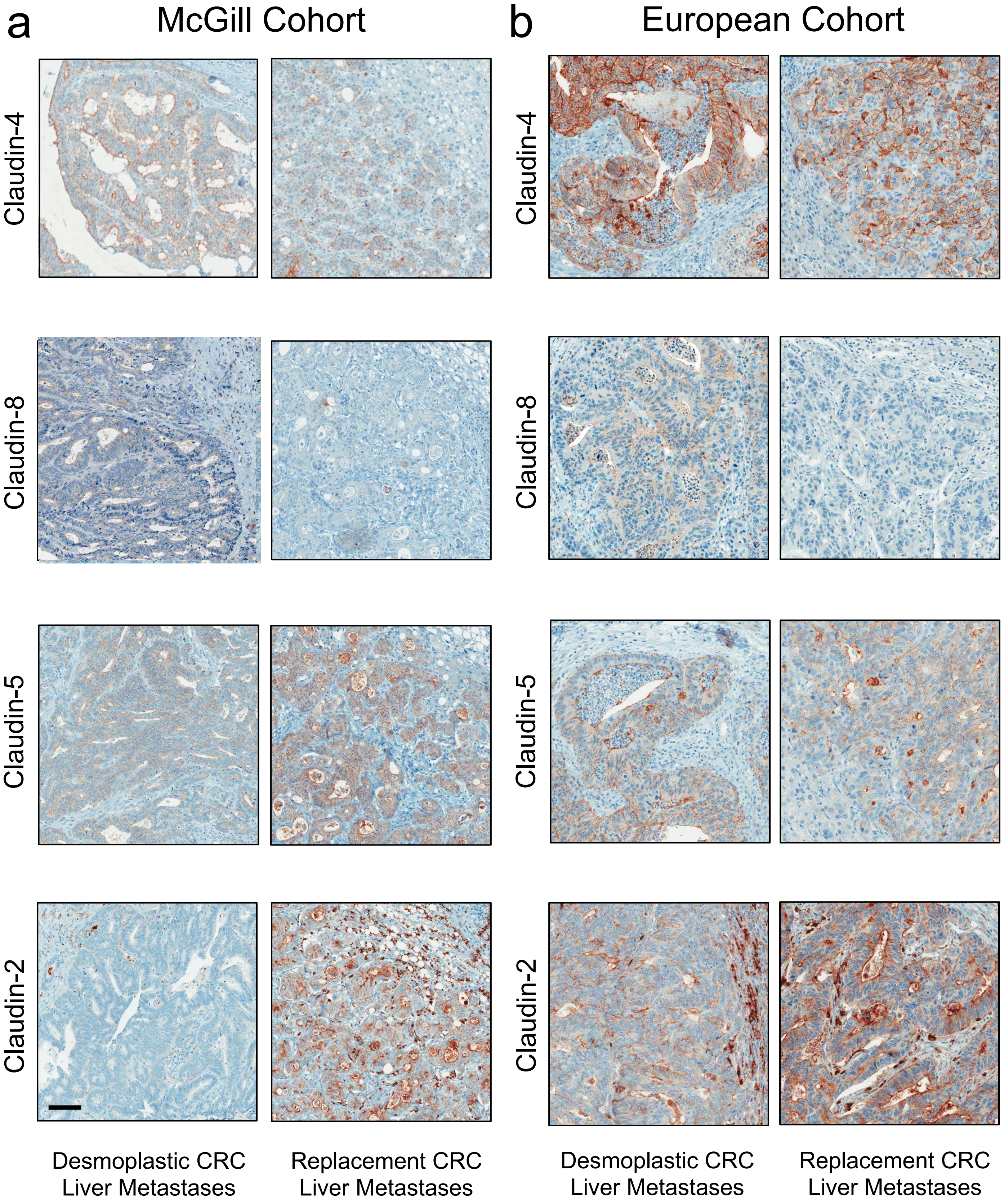

Tabariès *et al.*, Supplementary Figure 7

Supplementary Fig. 7: Claudin-2 protein is enriched in the replacement type lesions while higher Claudin-8 expression is associated with desmoplastic type liver metastases. a, b Representative IHC images from Claudin-4, Claudin-8, Claudin-5 or Claudin-2 staining are shown for both the McGill (**a**) and European (**b**) cohorts. Scale bar = 50 µm and applies to all panels. This figure is associated with Figure 4.

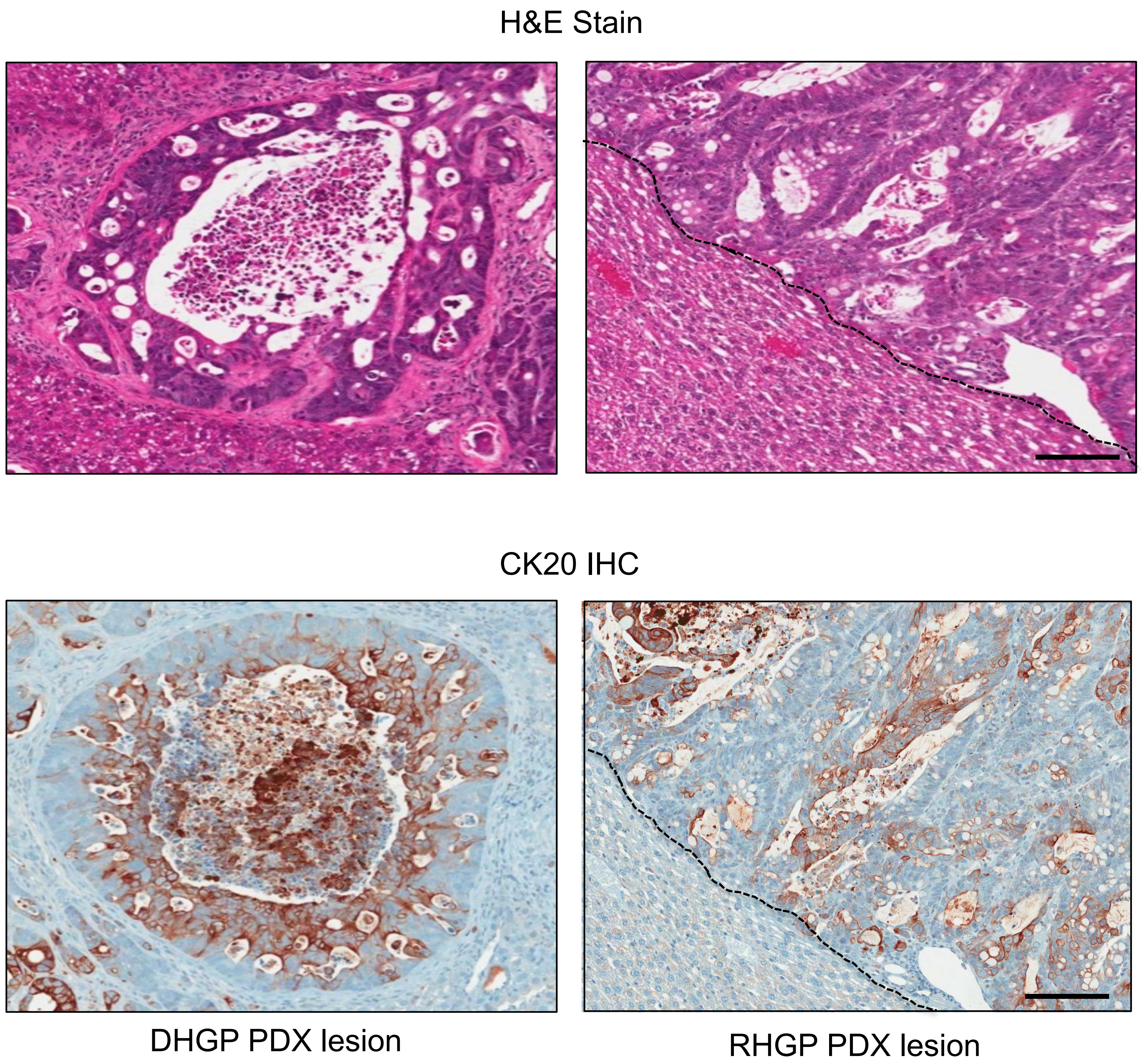

Tabariès *et al.*, Supplementary Figure 8

Supplementary Fig. 8: CK20 is expressed in PDXs models of both desmoplastic and replacement type colorectal cancer liver metastases. Paraffin embedded sections from DHGP or RHGP PDXs lesions were stained with H&E (*upper panels*) or subjected to immunohistochemical staining with anti-human specific CK20 antibody (*lower panels*). Scale bar = 50 μm and applies to all panels.

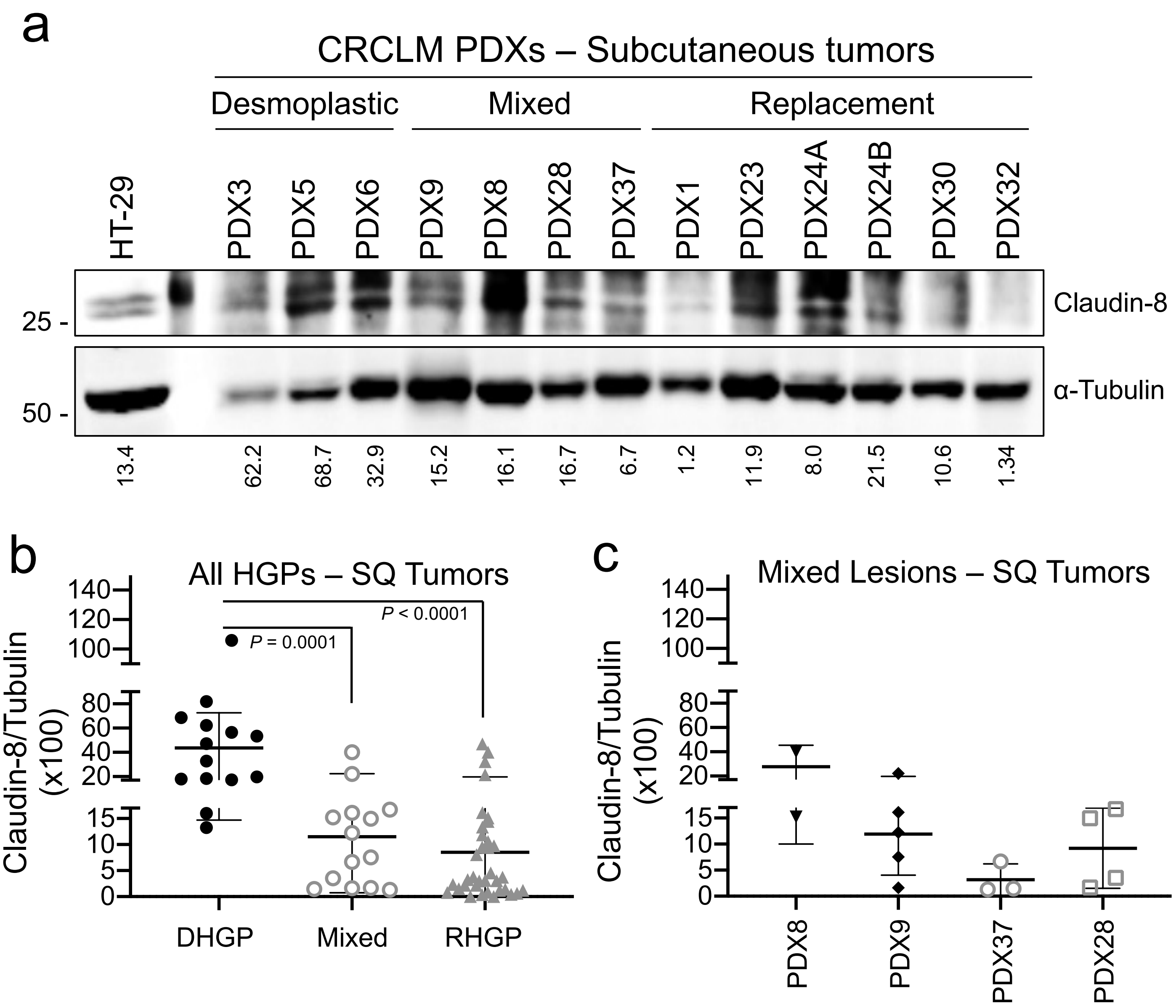

Tabariès *et al.*, Supplementary Figure 9

Supplementary Fig. 9: PDX-derived models for both replacement and desmoplastic type liver metastases. **a** Representative immunoblot analysis of Claudin-8 expression in subcutaneous tumor lysates from PDXs. As a loading control, total cell lysates were blotted for α-Tubulin. **b** Claudin-8 expression is elevated in sub-cutaneous tumors derived from desmoplastic type metastases. **c** Detailed assessment of the Claudin-8/Tubulin ratio in the mixed lesions. The ratio of Claudin-8 to α-Tubulin was measured using an Odyssey infrared imaging system and are indicated in each panel (**a-c**). Data are presented as the mean ± SE.

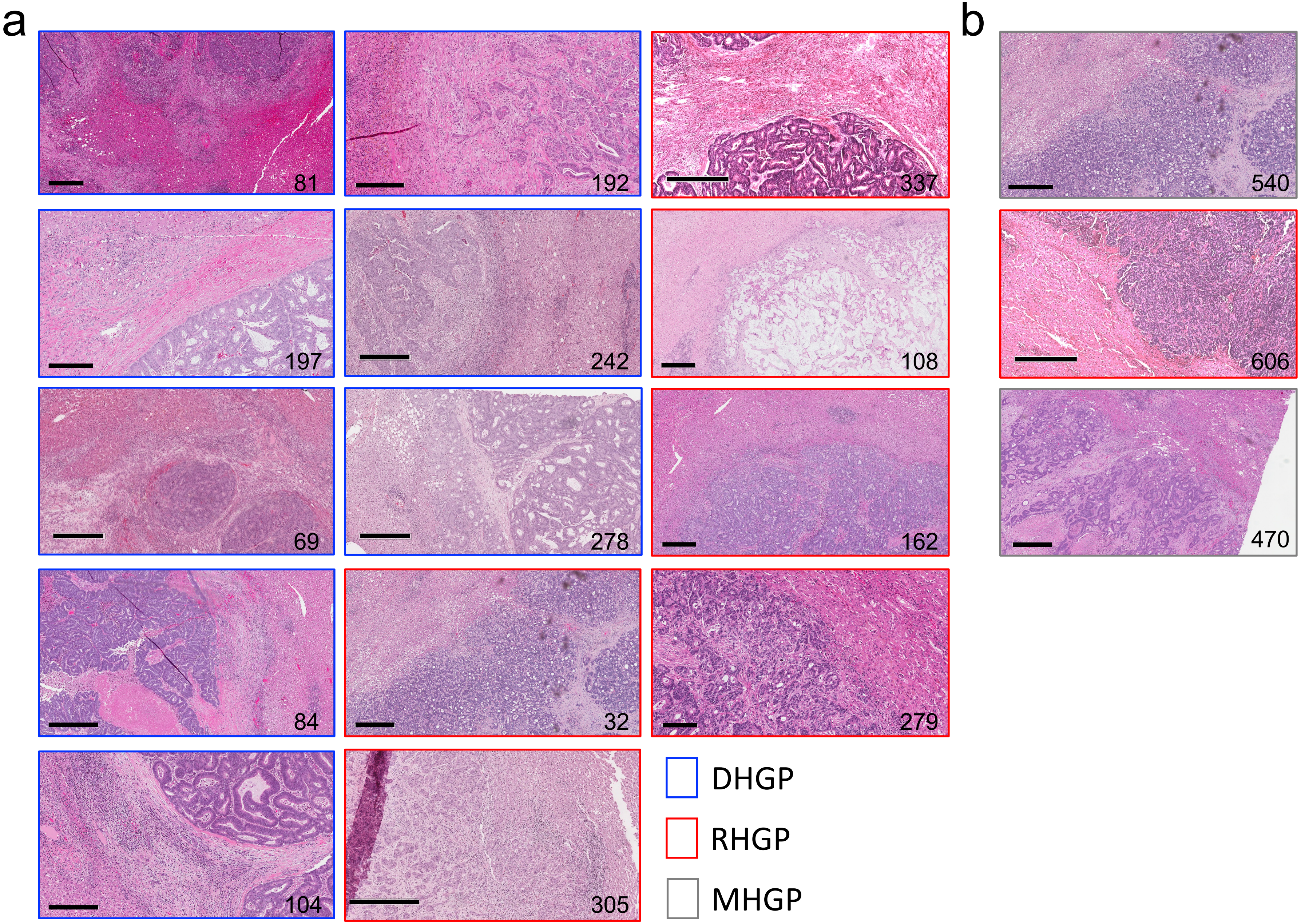

Tabariès *et al.*, Supplementary Figure 10

Supplementary Fig. 10: Histopathological growth patterns of colorectal cancer liver metastases from patients used for EV isolation. **a, b** DHGP or RHGP lesions paraffin embedded sections from patient from whom concentrated EVs samples were used in Figure 6b, c (**a**) or Figure 6 d, e (**b**) were stained with H&E. blue box outline = DHGP, red box outline = RHGP and grey box outline = mixed lesion (MHGP). Scale bar = 500 µm and applies to all panels.

Supplementary Table 1: Significance levels for CLDN2 expression in association with cancer gene mutations

	Gene	#Mutated Samples	log2(M/WT)	SE	P-Value
1	APC	239	-0.203	0.288	0.481
2	TP53	196	-0.180	0.278	0.516
3	TTN	160	-0.144	0.281	0.608
4	**KRAS**	**139**	**1.388**	**0.279**	**<0.0001**
5	**PIK3CA**	**104**	**1.420**	**0.302**	**<0.0001**
6	MUC16	95	0.093	0.320	0.772
7	SYNE1	90	0.017	0.326	0.958
8	FAT4	78	0.244	0.343	0.477
9	OBSCN	67	0.605	0.362	0.096
10	ZFHX4	67	-0.088	0.363	0.809
11	DNAH5	66	-0.604	0.364	0.098
12	RYR2	66	0.216	0.365	0.555
13	CSMD1	57	0.350	0.387	0.366
14	FLG	57	0.206	0.387	0.595
15	LRP1B	56	-0.144	0.390	0.712
16	PCLO	56	0.638	0.389	0.102
17	FAT3	55	0.407	0.393	0.301
18	CSMD3	54	-0.086	0.396	0.829
19	DNAH11	54	0.176	0.396	0.657
20	USH2A	53	0.194	0.399	0.627
21	FBXW7	52	-0.052	0.403	0.898
22	ABCA13	51	-0.310	0.406	0.446
23	RYR1	51	-0.166	0.406	0.682
24	HYDIN	50	-0.117	0.409	0.775
25	RYR3	50	-0.624	0.408	0.127
26	SDK1	50	-0.111	0.409	0.786

Supplementary Table 2: Patient-Derived Xenograft Models

RHGP[a] donors

PDX#	Gender	Age	Donor Patient HGP[c] Desmo.	Replac.	Pushing	Sub-cutaneous transplantation	Intra-hepatic Transplantation	PDX HGP
PDX1	Male	39	0	100	0	Succeed[d]	*Failed*[e]	N/A[f]
PDX11	Male	60	0	100	0	Not tested	Not tested	N/A
PDX23	Female	66	0	100	0	Succeed	Succeed	RHGP
PDX24A	Male	54	10	90	0	Succeed	Succeed	RHGP
PDX24B	Male	54	0	100	0	Succeed	Succeed	RHGP
PDX30	Female	85	0	100	0	Succeed	Succeed	RHGP
PDX32	Male	42	0	100	0	Succeed	Not tested	N/A
PDX33	Male	79	0	100	0	Succeed	Succeed	RHGP
PDX36	Male	42	0	100	0	Succeed	Failed	N/A
PDX37	Male	73	0	75	25	Succeed	Succeed	RHGP
PDX39	Male	64	5	80	15	*Failed*	*Failed*	N/A
PDX41	Female	48	0	100	0	Succeed	Succeed	RHGP
PDX42	Female	63	5	90	5	Succeed	Not tested	N/A
PDX62	Male	77	5	95	0	*Failed*	Not tested	N/A
PDX67	Female	73	0	100	0	Not tested	Not tested	N/A
PDX68	Female	67	5	95	0	Succeed	Succeed	RHGP
					success rate[g]	12/14 (86%)	8/11(73%)	

DHGP[b] donors

PDX#	Gender	Age	Donor Patient HGP Desmo.	Replac.	Pushing	Sub-cutaneous transplantation	Intra-hepatic Transplantation	PDX HGP
PDX3	Male	59	95	5	0	Succeed	Succeed	DHGP
PDX5	Male	58	95	5	0	Succeed	Succeed	DHGP
PDX6	Male	60	95	5	0	Succeed	Succeed	DHGP
PDX10	Male	68	100	0	0	Not tested	Not tested	N/A
PDX14	Male	60	100	0	0	*Failed*	*Failed*	N/A
PDX15	Female	49	100	0	0	*Failed*	*Failed*	N/A
PDX26B	Female	66	80	20	0	Not tested	Not tested	N/A
PDX35	Male	50	100	0	0	Succeed	Succeed	DHGP
PDX44	Male	50	100	0	0	Succeed	Succeed	DHGP
PDX48	Male	50	100	0	0	Succeed	Not tested	N/A
PDX50	Female	59	100	0	0	*Failed*	Not tested	N/A
PDX53	Male	80	95	5	0	Succeed	Succeed	DHGP
PDX54	Male	56	90	10	0	*Failed*	Succeed	DHGP
PDX57	Male	71	95	5	0	Not tested	Not tested	N/A
PDX64	Male	49	100	0	0	Not tested	Not tested	N/A
PDX65	Male	60	75	25	0	Not tested	Not tested	N/A
					success rate	7/11 (64%)	7/9 (78%)	

Mixed HGP donors

PDX#	Gender	Age	Donor Patient HGP Desmo.	Replac.	Pushing	Sub-cutaneous transplantation	Intra-hepatic Transplantation	PDX HGP
PDX4	Female	64	0	35	65	Succeed	Not tested	N/A
PDX8	Male	66	50	50	0	Succeed	Succeed	DHGP
PDX12	Male	39	10	30	60	*Failed*	*Failed*	N/A
PDX28	Male	41	40	55	5	Succeed	Succeed	DHGP
PDX43	Female	63	50	50	0	*Failed*	Not tested	N/A
PDX47	Male	43	30	70	0	Succeed	Succeed	RHGP/DHGP
PDX66	Male	62	45	35	20	Succeed	Succeed	RHGP/DHGP
PDX69	Male	76	65	30	5	Not tested	Not tested	N/A
					success rate	5/7 (71%)	4/5 (80%)	

Pushing HGP donors

PDX#	Gender	Age	Donor Patient HGP Desmo.	Replac.	Pushing	Sub-cutaneous transplantation	Intra-hepatic Transplantation	PDX HGP
PDX9	Male	74	0	20	80	Succeed	Not tested	N/A
					success rate	1/1 (100%)	N/A	

CRCLM Biopsy donors

PDX#	Gender	Age	Donor Patient HGP Desmo.	Replac.	Pushing	Sub-cutaneous transplantation	Intra-hepatic Transplantation	PDX HGP
PDX38	Male	59		N/A		Succeed	Succeed	RHGP
PDX40	Female	57		N/A		*Failed*	*Failed*	N/A
PDX45	Female	76		N/A		Succeed	Succeed	RHGP
PDX46	Female	44		N/A		Succeed	Succeed	DHGP
PDX49	Male	72		N/A		*Failed*	*Failed*	N/A
PDX51	Male	56		N/A		Not tested	Not tested	N/A
PDX56	Female	50		N/A		Not tested	Not tested	N/A
PDX58	Male	44		N/A		Not tested	Not tested	N/A
					success rate	3/5 (60%)	3/5 (60%)	

[a]RHGP: Replacement Histological Growth Pattern

[b]DHGP: Desmoplastic Histological Growth Pattern

[c]HGP: Histological Growth Pattern

[d]succeed: transplanted for at least 3 successive passages

[e]failed: no tumor growth

[f]N/A: Not applicable

[g]success rate: percentage of successful transplantation over tested samples

Supplementary Table 3: Patient-Derived EV samples

Patient ID	Gender	Age	Metastatic Sites	Liver Lesion size (cm)	Desmo.	Replac.	Pushing
81	Male	33	Liver	2.3	**90**	**10**	0
197	Male	77	Liver	9	**100**	0	0
79	male	68	Liver	1.2	**100**	0	0
84	Male	58	Liver	1.4	**90**	0	0
104	Male	69	Liver	5	**100**	0	0
192	Female	72	Liver	0.8	**95**	**5**	0
242	male	61	Liver	1.2	**100**	0	0
278	Female	78	Liver, Lung	1	**100**	0	0
204	Male	50	N/A	N/A	NA		
244	Male	40	N/A	N/A	NA		
32	Male	57	Liver, Lung	5.7	0	**100**	0
305	Female	63	Liver	5	0	**100**	0
337	Female	66	Liver	5.6	0	**100**	0
108	Female	56	Liver	9.7	**5**	**85**	**10**
162	Female	59	Liver	0.7	0	**100**	0
279	Male	75	Liver	1.8	**10**	**90**	0
540/540.1	Male	72	Liver	N/A	**70***	**30**	0
606/606.2	Female	80	Liver	N/A	0	**100**	0
464/464.2	Female	69	Liver, Lung	N/A	0	**100**	0
466/466.3	Female	45	Liver	N/A	**50**	**50**	0
470/470.2	Male	50	Liver, Lung	N/A	NA**		

* Mixed (MHGP) lesion as the main HGP was lower than 75% treshold

** Mixed (MHGP) lesion from which the relative contribution of desmoplastic vs replacement feature could not be ascribed

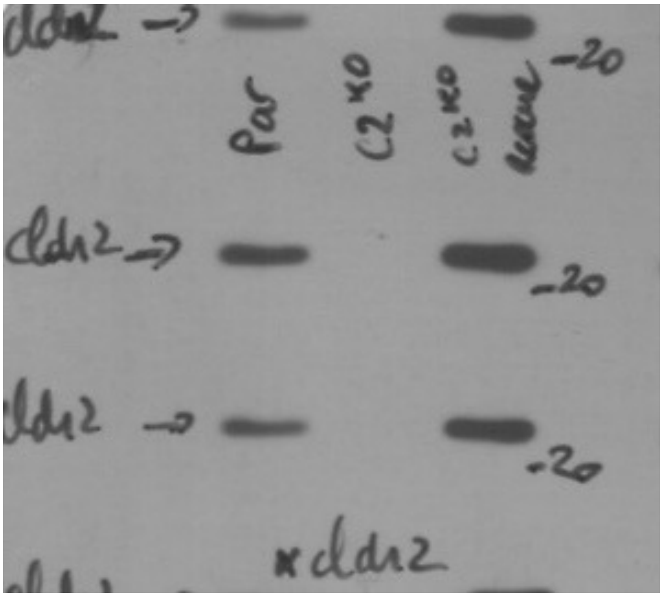

Exposure#1

Exposure#2

Exposure#3

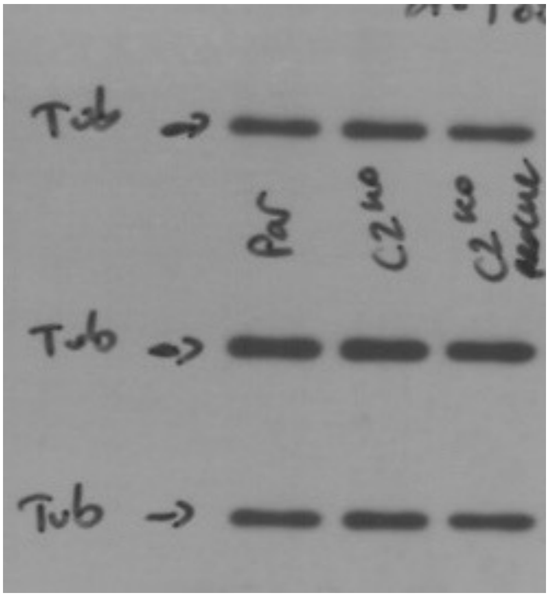

Exposure#1

Exposure#2

Exposure#3

Supplementary Figure 11 (Figure 1)

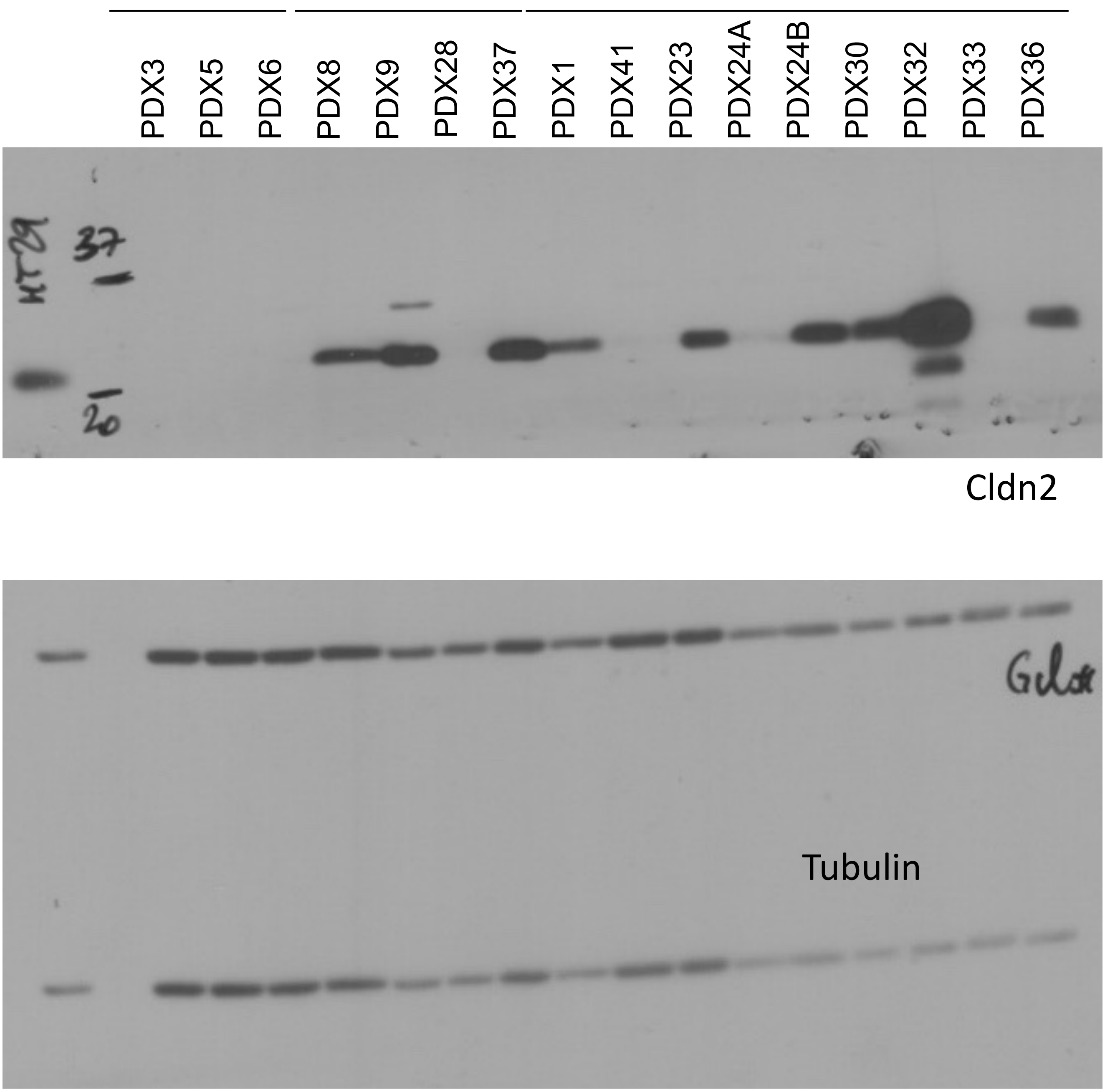

Supplementary Figure 11 (Figure 4)

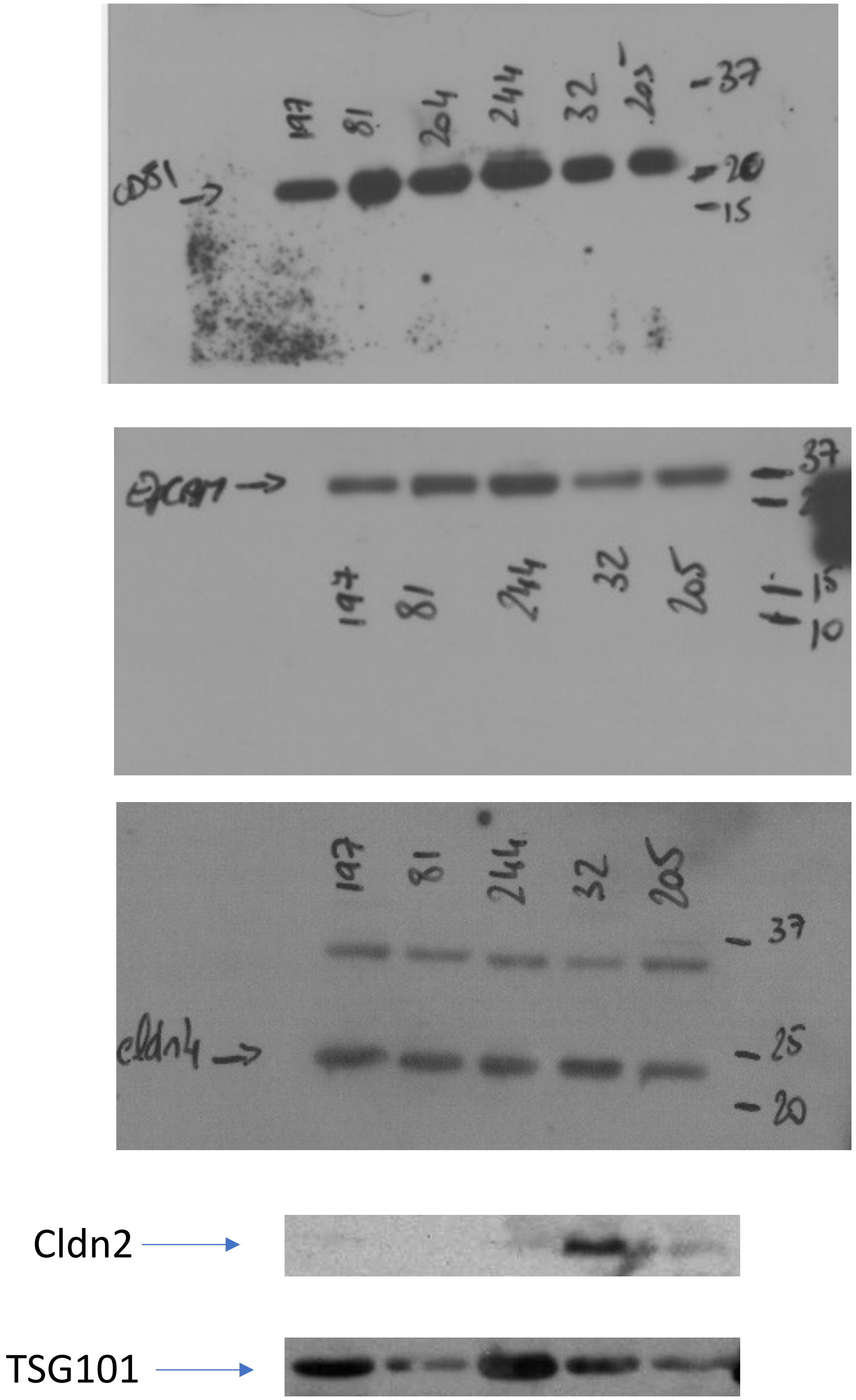

Supplementary Figure 11 (Figure 6)

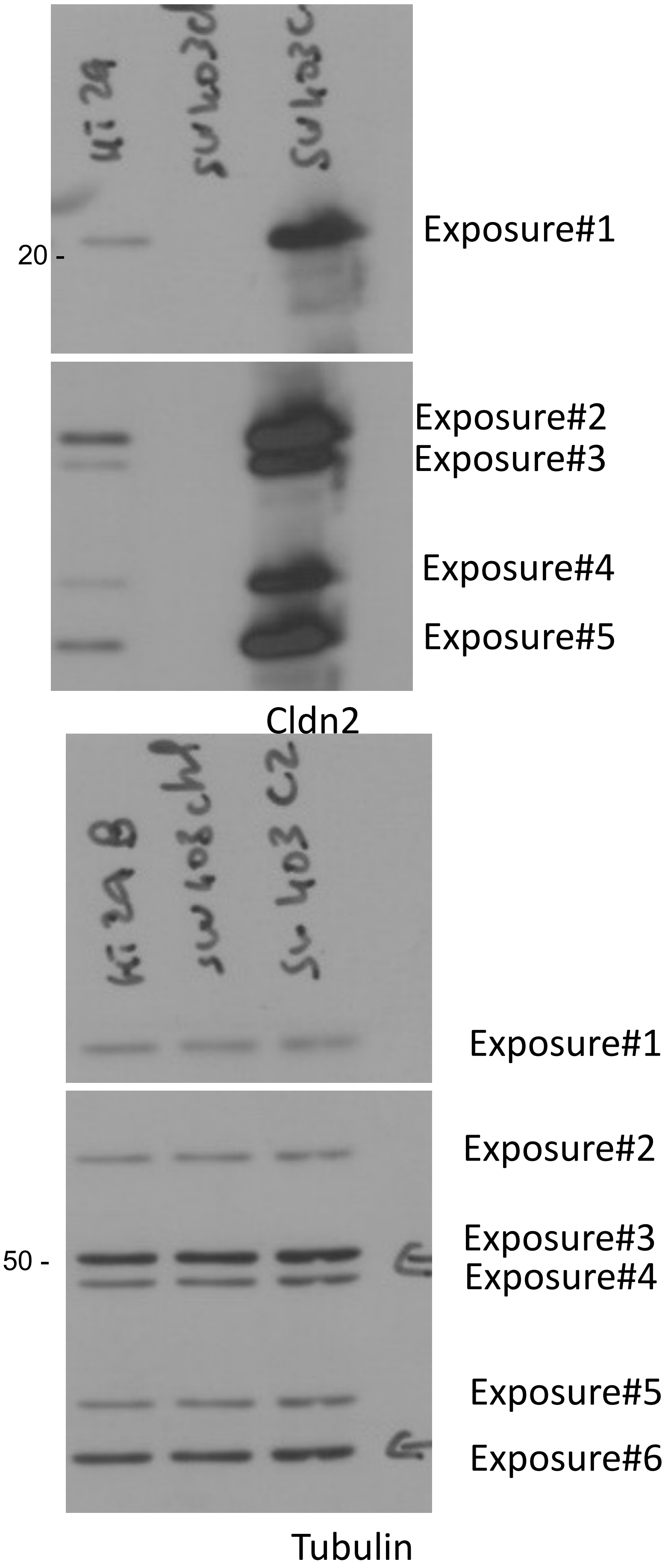

Supplementary Figure 11 (Figure S2)

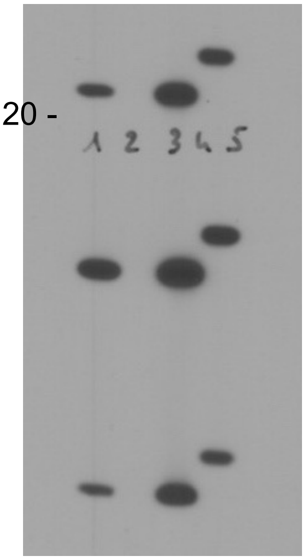

Cldn2

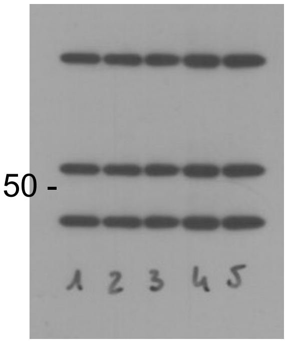

Tubulin

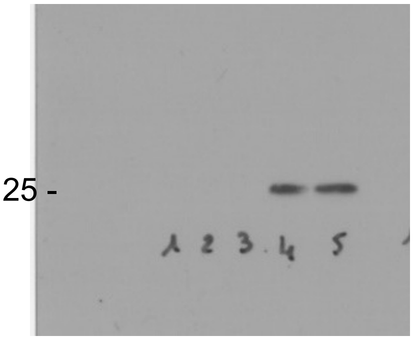

HA

1: HT29 parental
2: HT29: Cldn2^{KO}:EV
3: HT29: Cldn2^{KO}:WT
4: HT29: Cldn2^{KO}:WT (HA)
5: HT29: Cldn2^{KO}:ΔPDZ (HA)

Supplementary Figure 11 (Figure S3)

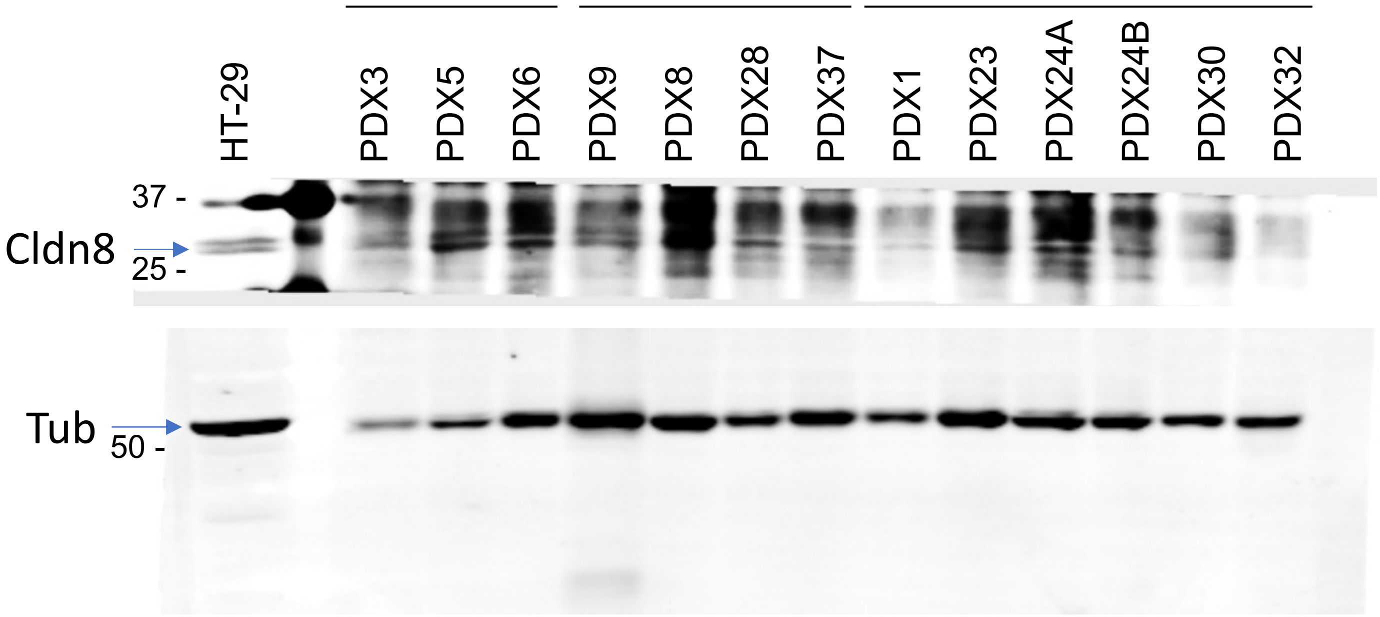

Supplementary Figure 11 (Figure S9)
