# Supplementary material for: Claudin-2 promotes colorectal cancer liver metastasis and is a biomarker of the replacement type growth pattern
Source: Commun Biol. 2021 Jun 2;4:657. doi: 10.1038/s42003-021-02189-9 (PMC8172859; doi:10.1038/s42003-021-02189-9)
Supplement: Supplementary file 2 — Description of Supplementary Files [file 42003_2021_2189_MOESM2_ESM.pdf]

Description of Additional Supplementary Files

File name: Supplementary Data 1

Description: Source data for Figures.
