# Supplementary material for: Claudin-2 promotes colorectal cancer liver metastasis and is a biomarker of the replacement type growth pattern
Source: Commun Biol. 2021 Jun 2;4:657. doi: 10.1038/s42003-021-02189-9 (PMC8172859; doi:10.1038/s42003-021-02189-9)
Supplement: Supplementary file 4 — Reporting Summary [file 42003_2021_2189_MOESM4_ESM.pdf]

nature research

Corresponding author(s): Dr. Peter Siegel - COMMSBIO-20-2199-B

Last updated by author(s): Apr 9, 2021

Reporting Summary

Nature Research wishes to improve the reproducibility of the work that we publish. This form provides structure for consistency and transparency in reporting. For further information on Nature Research policies, see our Editorial Policies and the Editorial Policy Checklist.

Statistics

For all statistical analyses, confirm that the following items are present in the figure legend, table legend, main text, or Methods section.

n/a	Confirmed	
☐	☒	The exact sample size (*n*) for each experimental group/condition, given as a discrete number and unit of measurement
☐	☒	A statement on whether measurements were taken from distinct samples or whether the same sample was measured repeatedly
☐	☒	The statistical test(s) used AND whether they are one- or two-sided<br>*Only common tests should be described solely by name; describe more complex techniques in the Methods section.*
☒	☐	A description of all covariates tested
☒	☐	A description of any assumptions or corrections, such as tests of normality and adjustment for multiple comparisons
☒	☐	A full description of the statistical parameters including central tendency (e.g. means) or other basic estimates (e.g. regression coefficient) AND variation (e.g. standard deviation) or associated estimates of uncertainty (e.g. confidence intervals)
☒	☐	For null hypothesis testing, the test statistic (e.g. *F*, *t*, *r*) with confidence intervals, effect sizes, degrees of freedom and *P* value noted<br>*Give P values as exact values whenever suitable.*
☒	☐	For Bayesian analysis, information on the choice of priors and Markov chain Monte Carlo settings
☒	☐	For hierarchical and complex designs, identification of the appropriate level for tests and full reporting of outcomes
☒	☐	Estimates of effect sizes (e.g. Cohen's *d*, Pearson's *r*), indicating how they were calculated

Our web collection on statistics for biologists contains articles on many of the points above.

Software and code

Policy information about availability of computer code

Data collection	Differential gene expression analysis was performed using DESeq2 (version 1.16.1) Bioconductor R package. The principal component analysis (PCA) was performed with the prcomp() R function and visualized with the pca3d (version 0.10) CRAN R package. Heatmaps were generated with heatmap.2 from gplots (version 3.0.1) R package. Gene expression (RSEM values) and mutation data for 18,939 genes from 377 colon adenocarcinoma samples (TCGA PanCancer Atlas) were downloaded from the cBioPortal for cancer genomics (https://www.cbioportal.org) using the R-package cgdsr (v1.3.0). Molecular subtypes (i.e. CMS, CRIS and MSI) were determined using the BioConductor package CMScaller (v0.99.2) based on the log2-transformed RSEM values.
Data analysis	Prism (Graphpad) v8.4.3 was used; Imagescope (Aperio) v10.0.36.1805

For manuscripts utilizing custom algorithms or software that are central to the research but not yet described in published literature, software must be made available to editors and reviewers. We strongly encourage code deposition in a community repository (e.g. GitHub). See the Nature Research guidelines for submitting code & software for further information.

Data

Policy information about availability of data

All manuscripts must include a data availability statement. This statement should provide the following information, where applicable:

- Accession codes, unique identifiers, or web links for publicly available datasets
- A list of figures that have associated raw data
- A description of any restrictions on data availability

RNAseq data used in Figure 2 has been made available publicly in GEO (GSE151165)

Field-specific reporting

Please select the one below that is the best fit for your research. If you are not sure, read the appropriate sections before making your selection.

☒ Life sciences ☐ Behavioural & social sciences ☐ Ecological, evolutionary & environmental sciences

For a reference copy of the document with all sections, see nature.com/documents/nr-reporting-summary-flat.pdf

Life sciences study design

All studies must disclose on these points even when the disclosure is negative.

Sample size	Previous studies from our laboratory have shown that a minimum of 8 animals per cohort is enough to assess liver metastasis burden. For all experiments involving human samples or human-derived samples, we have use the maximum number of samples we have able to access that would fit our requirements
Data exclusions	No data were excluded from the analyses
Replication	For experimental assays, cohorts were divided in two sets of injection to verify reproducibility.<br>For human samples, specimens were obtained from at least 2 independent sources (MUHC and OTB; McGill cohort and European cohort)
Randomization	Allocation was not random. Samples were allocated based on the liver metastasis histological growth pattern as assessed by pathologists
Blinding	Investigators were blinded to the histological growth pattern before histological staining and scoring

Reporting for specific materials, systems and methods

We require information from authors about some types of materials, experimental systems and methods used in many studies. Here, indicate whether each material, system or method listed is relevant to your study. If you are not sure if a list item applies to your research, read the appropriate section before selecting a response.

Materials & experimental systems

n/a	Involved in the study
☐	☒ Antibodies
☐	☒ Eukaryotic cell lines
☒	☐ Palaeontology and archaeology
☐	☒ Animals and other organisms
☐	☒ Human research participants
☐	☒ Clinical data
☒	☐ Dual use research of concern

Methods

n/a	Involved in the study
☒	☐ ChIP-seq
☒	☐ Flow cytometry
☒	☐ MRI-based neuroimaging

Antibodies

Antibodies used	the following antibodies were used: Claudin-2 (1:5,000;Cat. #: 325600, Thermofisher), Claudin-4 (1:5,000; Cat. #: 329400, Thermofisher), TSG101 (1:2,000; Cat. #: ab83, abcam), α-Tubulin (1:10,000; Cat. #: T9026, Sigma), and HA.11 clone 16B12 (1:10,000; Cat. #: MMS-101-P-200, Covance); Claudin-5 (1:300; Cat. #: 341600, Thermofisher Scientific), Claudin-8 (1:50; Cat. #: 400700Z, Thermofisher Scientific) and CK20 (1:200; Cat. #: 790-4431, Roche)
Validation	Antibodies against Claudin-2 and -4 have been widely used in our laboratory over the last year as well as α-Tubulin (PMID: 30692208; 25823815; 22645303; 21076473; 24287398). TSG101 is a well established marker for extracellular vesicle (32446904; 31390267; 26254847). There are more than 100 citations using the HA.11 antibodies for similar application. The use of the Claudin-5 has been cites 42 times for western blots and 21 times for immunohistochemical staining while the Claudin-8 antibody has been reported 2 times for western blots and 6 times for immunohistochemical staining. Finally, the CK20 antibody has been widely used for immunohistochemical staining using the ventana system (Roche) as we did.

Eukaryotic cell lines

Policy information about cell lines

Cell line source(s)	The HT-29 and SW403 cell lines were obtained from the American Type CultureCollection (ATCC).
Authentication	The cell lines used were authenticated by vendor (ATCC).
Mycoplasma contamination	All cell lines were tested negative for mycoplasma (MycoAlert Mycoplasma Detection Kit; Lonza)

Commonly misidentified lines (See ICLAC register)	N/A

Animals and other organisms

Policy information about studies involving animals; ARRIVE guidelines recommended for reporting animal research

Laboratory animals	4- to 6-week-old female Scid/beige were used
Wild animals	The study did not involve wild animal
Field-collected samples	The study did not involve samples collected from the field
Ethics oversight	All animal experiments were conducted under a McGill University approved Animal Use Protocol in accordance with guidelines established by the Canadian Council on Animal Care.

Note that full information on the approval of the study protocol must also be provided in the manuscript.

Human research participants

Policy information about studies involving human research participants

Population characteristics	This study included a total of 97 CRC primary tumors either from the Liver Disease Biobank (LBD; McGill University) or from Ontario Tumor Bank. Matched resected liver metastatic lesions from 22 chemo-naïve patients, representing 23 chemo-naive lesions (8 DHGP and 15 RHGP), were also obtained from the LBD.<br>I n addition, FFPE samples of 64 CRCLM resection specimens were obtained from the biobank of the GZA Hospital Sint-Augustinus, Antwerp, Belgium.
Recruitment	Informed consent was obtained from all patients through the MUHC Liver Disease Biobank Surgical specimens were procured and released to the Biobank immediately after the pathologist's confirmation of carcinoma and surgical margins. Biological materials were also provided by the Ontario Tumour Bank (OTB), which is funded by the Ontario Institute for Cancer Research (OICR).<br>For the European cohort, the samples were entered into the biobank after written informed consent of patients in the Sint-Augustinus Hospital, Antwerp, Belgium (n=39) and in the Erasmus MC Cancer Institute, Rotterdam, The Netherlands (n=25). Standard peri-operative systemic treatment was given in 31 patients.
Ethics oversight	LDB: MUHC research ethics board approved protocol SDR-11-066<br>European cohort: Federal Agency Notification Number BB190028 obtained after approval by the local ethics review board

Note that full information on the approval of the study protocol must also be provided in the manuscript.

Clinical data

Policy information about clinical studies

All manuscripts should comply with the ICMJE guidelines for publication of clinical research and a completed CONSORT checklist must be included with all submissions.

Clinical trial registration	N/A
Study protocol	MUHC research ethics board approved protocol SDR-11-066 for LDB.<br>The Ontario Tumour Bank (OTB) provides cancer researchers with a diverse selection of high-quality biospecimens and derivatives, comprehensively annotated with de-identified clinical data.
Data collection	MUHC research ethics board approved protocol SDR-11-066 for LDB.<br>The Ontario Tumour Bank (OTB) provides cancer researchers with a diverse selection of high-quality biospecimens and derivatives, comprehensively annotated with de-identified clinical data.
Outcomes	5-years overall survival and 5-years Relapse Free Survival
